# Inter-Organelle Membrane Contact Sites as Physiological Regulatory Hubs in the Brain: From Neurons to Glial Cells

**DOI:** 10.3390/biom16091257

**Published:** 2026-08-31

**Authors:** Yuchen Wu, Ginam Cho, Youngshin Lim

**Affiliations:** 1 Department of Pathology and Laboratory Medicine, Cedars-Sinai Health Sciences University, Los Angeles, CA 90048, USA; yuchen.wu@cshs.org (Y.W.);; 2 School of Medicine, Tsinghua Medicine, Tsinghua University, Beijing 100084, China; 3 Department of Biomedical Science Education, College of Medicine, Charles R Drew University of Medicine and Science, Los Angeles, CA 90059, USA

**Keywords:** membrane contact sites, neurons, glial cells, organelle interactions, neurological diseases, brain homeostasis, potential therapies

## Abstract

Inter-organelle membrane contact sites (MCSs) enable direct communication between organelles, and this communication is fundamental to cellular homeostasis, coordinated calcium (Ca^2+^) signaling, lipid metabolism, energy production, and stress responses. While MCSs are evolutionarily conserved, emerging evidence indicates that their organization and function are highly context dependent. In the brain, neurons and glial cells differ markedly in their physiological roles, morphologies, and metabolic demands, suggesting that inter-organelle contact networks might be specialized in a cell-type-dependent manner. Although much of the existing literature focuses on neurons, growing evidence indicates that these contact sites also play important roles in glial cells. Here, we review recent advances in our understanding of MCSs in the nervous system, focusing on cell-type-specific differences between neurons and glial cells. We highlight the spatial specialization of MCSs within neurons, emphasizing how subcellular localization shapes their functional output. Our analysis of the current literature suggests that neuronal MCSs are primarily optimized for rapid Ca^2+^ signaling and metabolic adaptation, whereas glial MCSs preferentially coordinate lipid metabolism, inflammatory signaling, and tissue homeostasis. We also review the context-dependent and disease-driven remodeling of MCSs in the brain, reflecting alterations in contact-site composition and function rather than simply increased or decreased organelle proximity. Furthermore, we discuss emerging therapeutic perspectives aimed at modulating inter-organelle communication in multiple neurological diseases and outline key unresolved questions and future directions necessary to elucidate how inter-organelle contact sites shape brain physiology and disease. Collectively, the evidence reviewed here indicates that MCSs serve as dynamic signaling platforms, with their specific physiological and pathological functions varying according to cell type, subcellular localization, and molecular composition.

## 1. Introduction

Eukaryotic cells rely on complex communication between membrane-bound organelles to coordinate metabolism, signaling, and stress responses. Beyond vesicular trafficking, this communication is largely mediated by membrane contact sites (MCSs). MCSs are defined as regions where the membranes of two or more organelles are closely apposed without membrane fusion [1]. Evolutionarily conserved [2] and found between almost all kinds of organelles, these sites are characterized by an intermembrane distance typically ranging from 10 to 80 nm, with an average spacing of approximately 30 nm [3]. At these sites, organelles exchange ions, lipids, and metabolites, while organizing signaling complexes with high spatial and temporal precision [4,5].

Among the various MCSs, those formed by mitochondria have been intensively studied. For instance, endoplasmic reticulum (ER)-mitochondria contact sites, often referred to as mitochondria-associated membranes (MAMs), regulate Ca^2+^ transfer, lipid biosynthesis, mitochondrial dynamics, and stress signaling [6,7,8,9,10]. Crosstalk between lysosomes and mitochondria, referred to as mitochondria-lysosome contact sites, is increasingly recognized for its function in the maintenance of neural homeostasis [11]. Mitochondrial contact sites with the nucleus are much less well described, yet they play an important role in metabolite exchange and signal transduction between the two organelles [12]. Dysregulation of mitochondria-based MCSs has been implicated in a broad range of pathological conditions, including metabolic disorders, cancer, and neurodegenerative diseases. However, much of our current understanding of MCS biology derives from studies in non-neuronal or immortalized cell lines, which may not be directly applicable to neurons or other cell types in the brain.

Cells in the brain can be roughly divided into two types: neurons and glial cells. Although present in the same system, they exhibit distinct morphologies, metabolic demands, and physiological roles. Neurons are excitable cells that depend on rapid and spatially restricted Ca^2+^ signaling, continuous ATP supply, and long-distance organelle transport, which support synaptic transmission and plasticity [13]. In contrast, glial cells are metabolically versatile cells that play central roles in lipid metabolism, neurotransmitter recycling, immune surveillance, and inflammatory signaling [14]. These profound differences raise a question: do neurons and glial cells have the same membrane contact sites, or are MCSs strongly influenced by cellular context within the brain?

Recent advances in high-resolution imaging, proximity labeling, and genetically encoded reporters [15] have revealed pronounced cell-type and context-dependent differences in inter-organelle interactions. Neurons display extensive endoplasmic reticulum-mitochondria coupling at dendrites and synapses, supporting activity-dependent Ca^2+^ transfer and metabolic adaptation, whereas glial cells preferentially organize lysosome-centered contact networks linked to lipid handling, degradation, and immune signaling [16]. Moreover, emerging data indicate that the composition, dynamics, and even biophysical properties of MCSs can differ substantially between neuronal and glial compartments. Cell-type-dependent inter-organelle interactions have significant implications for multiple disease conditions. For example, alterations in mitochondria-associated contacts have been reported in neurodegenerative disease, excitotoxic injury, and neuroinflammation. In these conditions, the changes in contact density, tethering composition, and MCS dynamics are frequently accompanied by alterations in Ca^2+^ signaling, lipid exchange and mitochondrial quality control. These changes often occur in a cell-type-specific manner and contribute to distinct pathological outcomes depending on the physiological roles of individual neural cell types [17,18,19,20,21,22]. In microglia, pathological remodeling of MCSs has been linked to aberrant inflammasome activation and dysregulated innate immune signaling [21,23,24], whereas in neurons, dysregulation of MCSs contributes to Ca^2+^ signaling dysregulation and lipid trafficking deficits, leading to mitochondrial dysfunction and synaptic failure [25]. These observations suggest that disease states may reveal the functional logic underlying MCS specialization in different brain cell types.

In this review, we provide an overview of current knowledge on membrane contact sites in the brain with a focus on their cell-type-specific organization and function. We compare the molecular architecture, spatial distribution, and functional outputs of MCSs in neurons and glial cells and discuss how these specialized contact networks support distinct physiological roles and are altered in multiple disease conditions. Finally, we highlight key open questions and emerging concepts, including dynamic remodeling, phase separation, and therapeutic targeting of pathological MCSs, aiming to provide a conceptual framework for understanding inter-organelle communication in the nervous system.

## 2. Architectural Specialization of MCSs in Neurons and Glial Cells

### 2.1. Neuronal MCSs: Mitochondria-Centered and Activity-Coupled Networks

**Figure 1:**
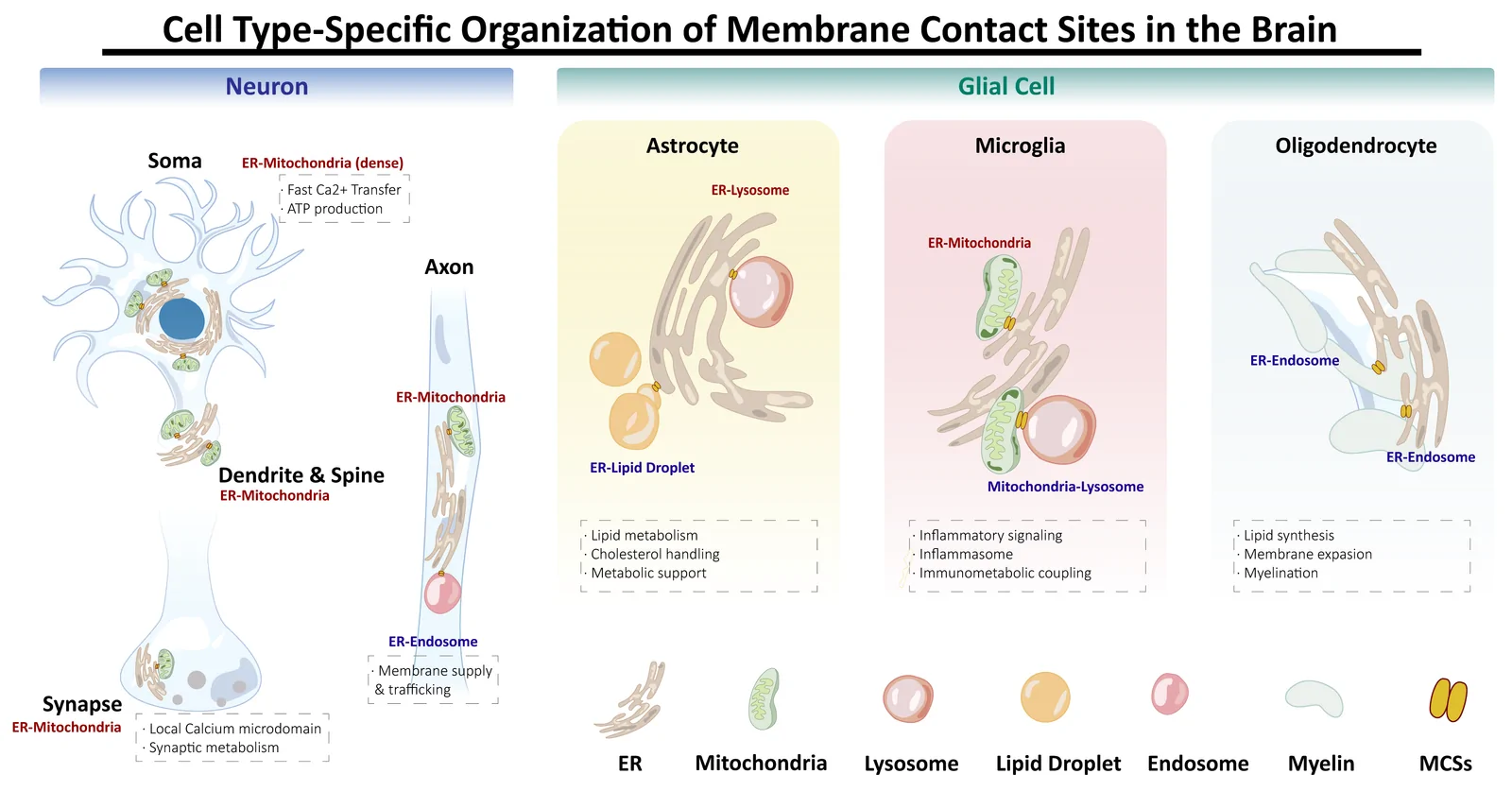
Cell type-specific organization of membrane contact sites in the brain. Neurons and glial cells organize membrane contact sites (MCSs) according to their functional demands. Neuronal MCS networks are centered on ER–mitochondria interfaces that support rapid Ca^2+^ transfer, activity-dependent ATP production, and synaptic metabolism across soma, dendrites, and synapses. In contrast, glial cells exhibit lysosome- and lipid-associated MCSs. Astrocytes engage ER–lysosome and ER–lipid droplet contacts for lipid handling and metabolic support, microglia utilize ER–mitochondria and mitochondria–lysosome contacts for immune signaling, and oligodendrocytes form ER–plasma membrane and ER–myelin contacts to sustain myelin growth. These differences underscore that MCS organization is tailored to cell identity and function in the brain. Red words indicate membrane contact sites that are supported by extensive molecular and functional evidence, whereas blue words represent emerging or less-well characterized interactions, whose molecular composition and physiological significance remain under investigation.

Neurons are highly polarized cells with extreme demands for spatially restricted Ca^2+^ signaling, continuous ATP supply, and long-distance organelle transport [26,27,28,29,30,31]. Consistent with these requirements, membrane contact sites in neurons are prominently organized around mitochondria and the ER, forming dense ER-mitochondria contact networks that support rapid excitation-metabolism coupling [16]. Neurons have more abundant and closely spaced MAMs compared to astrocytes, while astrocytes have more lysosome-related contact sites [16]. Accordingly, contact sites involving each cell type’s dominant ‘hub’ organelles appear more prevalent and functionally emphasized (ER- or mitochondria-centered contacts in neurons and lysosome-centered contacts in astrocytes), consistent with their distinct physiological priorities [16] (Figure 1).

Well-characterized tethering complexes at neuronal ER-mitochondria contact sites include the IP3R-GRP75-VDAC axis. The Inositol 1,4,5-trisphosphate receptors (IP3Rs) on the ER membrane are physically and functionally coupled to VDAC1 (voltage-dependent anion channel 1) on the outer mitochondrial membrane through the molecular chaperone GRP75 (glucose-regulated protein 75 kDa), forming a tethering complex that enables efficient ER-to-mitochondria Ca^2+^ transfer at contact sites [32]. This axis enables efficient Ca^2+^ transfer from the ER lumen to the mitochondrial matrix [2,3,33], forming a microdomain that allows mitochondria to selectively sense Ca^2+^ signals without disrupting Ca^2+^ levels throughout the cytosol. This tethering system is particularly well-suited to couple activity-dependent ER Ca^2+^ release to mitochondrial ATP production in dendrites and synaptic compartments in neurons [31], where energy demand changes rapidly during neurotransmission.

Another well-characterized ER-mitochondria tether in neurons is formed by the interaction between the ER-resident protein VAPB (vesicle-associated membrane protein-associated protein B) and the outer mitochondrial membrane protein PTPIP51 (protein tyrosine phosphatase-interacting protein 51) [34,35]. This tether plays a key role in maintaining ER-mitochondria proximity and dynamically regulating multiple signaling outputs. Genetic or biochemical manipulation of VAPB or PTPIP51 expression bidirectionally regulates the abundance of ER-mitochondria contacts: loss of either component reduces, whereas overexpression enhances, inter-organelle coupling [34,36]. Functionally, VAPB-PTPIP51-mediated contacts regulate both ER-to-mitochondria Ca^2+^ transfer and lipid metabolism, thus further influencing mitochondrial ATP production and autophagy [34,36,37,38,39]. PTPIP51 and VAPB are more highly expressed in neurons than in glial cells, consistent with neurons’ greater Ca^2+^ demand [16]. This tether additionally supports synaptic function by stabilizing mitochondria near dendritic spines and regulating synaptic vesicle cycling, thereby linking organelle positioning to synaptic plasticity [35,37,40].

The ER-resident tether PDZD8 (PDZ domain containing 8), a functional ortholog of the yeast ER-mitochondria contact site component Mmm1 (maintenance of mitochondrial morphology protein 1) [41], is expressed in neurons but absent from certain glial cell types [42], highlighting a clear cell type-specific deployment of this tethering machinery. Loss of PDZD8 disrupts ER-mitochondria proximity, leading to impaired mitochondrial Ca^2+^ buffering and altered dendritic Ca^2+^ dynamics, thereby directly linking PDZD8-dependent contact architecture to activity-coupled neuronal signaling [41]. Recent work has revealed a novel mechanism by which PDZD8 contributes to the formation and stabilization of inter-organelle contact sites [43]. PDZD8 contains an intrinsically disordered region (IDR) that enables liquid–liquid phase separation, leading to the formation of membrane-associated condensates both in vitro and in mammalian cell lines [43]. Ultrastructural analyses demonstrated that full-length PDZD8, but not IDR-deficient variants, restores ER-mitochondria contacts in PDZD8-deficient cells, indicating that phase separation is essential for its tethering function [43]. These findings suggest that PDZD8 condensates act as an adhesive scaffold at lipid interfaces, physically stitching adjacent organelles together, and thereby supporting the structural and functional integrity of ER-mitochondria communication [41].

Beyond these tethers and tethering complexes, mitochondrial dynamics proteins, such as mitofusin 2 (Mfn2), also link membrane contacts to neuronal physiology. In addition to its well-known function in mitochondrial fusion, Mfn2 also contributes to the formation and stability of ER-mitochondria and mitochondria-lipid droplet contact sites [44,45,46], a regulatory role that is also evident in neurons. Studies conducted in pyramidal neurons in vivo and neuroblastoma cells indicate that Mfn2 positively regulates ER–mitochondria coupling. In these systems, Mfn2 overexpression increases the formation of MAMs, enhances IP3R3–Grp75 interaction and mitochondrial Ca^2+^ uptake, and strengthens the association between the tethering pair VAPB and PTPIP51, whereas Mfn2 ablation produces the opposite effects [46]. However, the role of Mfn2 in MAM formation is not entirely straightforward. In vitro studies have reported that Mfn2 ablation or silencing can increase the number of close ER–mitochondria appositions and potentiate inositol 1,4,5-trisphosphate (IP_3_)-induced Ca^2+^ transfer from the ER to mitochondria [47]. Rather than simply arguing that Mfn2 is either an agonist or an antagonist, these apparently conflicting observations suggest a more nuanced model in which Mfn2 acts as a context-dependent regulator of contact architecture and function. Its effects may vary depending on cell type, experimental system, and the structural or functional parameters used to define ER–mitochondria contacts.

The importance of Mfn2 also extends beyond MCS morphology. In neurons, Mfn2 is required for proper cell physiology. Mechanistically, ER-localized Mfn2 interacts with mitochondrial mitofusins (Mfn1/2) to promote ER–mitochondria communication, thereby facilitating Ca^2+^ transfer from the ER to mitochondria and enhancing mitochondrial bioenergetics. The functional relevance of this mechanism is evident during neurite outgrowth, as an increase in Mfn2-dependent ER–mitochondria contacts is required to support the metabolic demands of neuronal arborization [48]. Studies using human induced pluripotent stem cell (hiPSC)-derived neuronal models have also shown that Mfn2 overexpression in neural progenitor cells promotes neuronal differentiation, accompanied by enhanced mitochondrial function, whereas Mfn2 knockdown leads to mitochondrial dysfunction and impaired neurogenesis and synapse formation [49].

While Mfn2 plays an important role in maintaining organelle and cell homeostasis, it is also dynamically regulated under pathological conditions. Excitotoxic insults trigger a biphasic mitochondrial fragmentation process, in which early dynamin-related protein 1 (DRP1)-dependent fission is followed by a delayed phase driven by transcriptional downregulation of Mfn2, leading to persistent mitochondrial dysfunction, Ca^2+^ dysregulation, and delayed neuronal death [20]. These findings position Mfn2 as a key node linking neuronal activity, mitochondrial dynamics, and MCS integrity, while emphasizing that its influence on contact sites is context dependent.

Collectively, neuronal MCSs can be viewed as fast, highly localized, and activity-responsive platforms centered on mitochondria. Current knowledge of neuronal MCSs largely comes from cortical and hippocampal neurons, so whether MCSs architecture and function differ across neuron populations (e.g., multipolar neurons, bipolar neurons, pseudounipolar neurons, unipolar neurons, etc.) remains unclear. Likewise, studies in yeast, Drosophila, together with mammalian systems, support the evolutionary conservation of core MCS functions and several key MCS regulators [50]. Their molecular composition and spatial organization are tuned to support synaptic signaling, metabolic flexibility, and long-term neuronal survival.

### 2.2. Glial MCSs: Lysosome- and Immune-Centered Interaction Hubs

In contrast to neurons, glial cells, including astrocytes, oligodendrocytes, and microglia, exhibit distinct metabolic strategies and functional priorities, which are reflected in the organization of their membrane contact sites. Rather than being dominated by mitochondria-centered Ca^2+^ signaling modules, glial MCSs prominently involve lysosomes, late endosomes, and the ER, supporting lipid metabolism, degradation pathways, and immune signaling [16] (Figure 1).

Astrocytes play central roles in lipid handling, cholesterol trafficking, metabolic coupling, and inflammatory monitoring in the brain, functions that rely heavily on membrane contact sites organized around the ER, lysosomes, and endolysosomal compartments. Contacts formed around lysosomes play a key role in coordinating cholesterol trafficking [51] and mechanistic target of rapamycin complex 1 (mTORC1) signaling, which are involved in astrocyte activation and neuroinflammatory responses [52,53,54]. At the ER-lysosome interface, oxysterol-binding protein (OSBP) functions with ER anchors VAPA (vesicle-associated membrane protein-associated protein A) and VAPB to mediate the transfer of cholesterol from the ER to the lysosome, thereby facilitating cholesterol-dependent mTORC1 activation through the Ras-related GTP-binding (Rag) GTPase pathway [55]. Such cholesterol flux and mTORC1 regulation are essential for astrocytic lipid handling and metabolic support functions. Beyond lysosome-associated sites, lipid droplets are typically located in the perinuclear region and are frequently positioned in close proximity to mitochondria and the ER [56], thereby promoting the transfer of fatty acids between these organelles, especially from lipid droplets to mitochondria for β-oxidation and ATP generation [57].

Astrocyte contact networks are highly dynamic and sensitive to pathological perturbation. Under physiological conditions, MAMs are preferentially enriched within perivascular endfeet in astrocytes, and they establish specialized metabolic domains supporting Ca^2+^ homeostasis, lipid metabolism, and neurovascular coupling [58]. At the ultrastructural level, these contact sites at perivascular endfeet maintain a mean separation distance of 18.9 ± 5.0 nm between two organelles. In contrast, mitochondria in perisynaptic astrocytic processes remain at a similar distance from the ER but are smaller, and the extent of the ER membrane is also reduced, resulting in significantly fewer contact sites [59]. Following injury, reactive astrocytes undergo a marked spatial remodeling of these organelles, which is characterized by perivascular mitochondrial clustering accompanied by enhanced ER–mitochondria tethering [60]. Studies indicate that Mfn2 is partially involved in this process [59]. These alterations promote mitochondrial Ca^2+^ buffering, vascular remodeling, and tissue repair [60]. However, whether reactive astrocytes can similarly remodel other classes of MCSs remains unclear. It is important to note that astrocytes derived from Alzheimer’s disease (AD) mouse models, as well as wild-type astrocytes acutely exposed to amyloid-β peptides, display a marked increase in ER-mitochondria juxtaposition [61], indicating that disease-associated stress can rewire the balance of astrocytic MCS architectures. Although the mechanisms underlying this response have not been directly investigated in astrocytes, studies in hippocampal neuronal models suggest that Aβ may enhance ER–mitochondria functional coupling by remodeling MAM-associated Ca^2+^-transfer machinery, particularly the IP3R–GRP75–VDAC1 axis, and by altering the expression of selected MAM-associated proteins (see Section 5.1). Nevertheless, this remodeling suggests a prominent involvement of mitochondria-centered signaling under pathological conditions, potentially reflecting altered Ca^2+^ handling, metabolic stress responses, or inflammatory activation.

Microglia, another type of glial cell, are the resident immune cells of the central nervous system and require efficient coordination of metabolic state, Ca^2+^ signaling, and pattern-recognition pathways mediated by pattern recognition receptors (PRRs) to support rapid immune surveillance. In microglia, MCSs are functionally specialized to organize immune-related signaling rather than activity-dependent bioenergetic coupling. In particular, MAMs act as spatial platforms that integrate mitochondrial Ca^2+^ handling, redox status, and lipid signaling with innate immune receptor complexes [23]. At these ER-mitochondria interfaces, MAMs facilitate the proximity-based assembly of immune signaling machinery by positioning NLRP3 (nucleotide-binding and oligomerization domain-like receptor family pyrin domain-containing 3) and its adaptor ASC (apoptosis-associated speck-like protein containing a caspase activation and recruitment domain) near mitochondria-derived danger signals [21]. This recruitment to MAMs positions inflammasome components at the interface of ER and mitochondria, enabling efficient signal integration [21].

Beyond MAMs, emerging evidence suggests that additional communication networks between organelles may also contribute to microglial homeostasis. Among them, mitochondria-lysosome membrane contact sites have recently gained attention as regulatory hubs. However, compared with ER-mitochondria contact sites, the molecular organization and function of mitochondria-lysosome contacts in microglia remain uncharacterized. Current mechanistic understanding is largely derived from non-glial systems. Studies in multiple cellular systems have demonstrated that the formation and dissociation of mitochondria-lysosome contact sites are modulated by the lysosomal small GTPase Ras-related protein Rab-7 (RAB7). The GTP-bound active form of RAB7 promotes organelle tethering, whereas contact release is driven by mitochondrial recruitment of the lysosomal protein RAB7 GTPase-activating protein TBC1D15 (TBC1 domain family member 15) via the outer mitochondrial membrane protein FIS1 (mitochondrial fission 1), thereby establishing a bidirectional regulatory loop between the two organelles [62,63]. However, direct evidence supporting their operation in microglia remains limited.

Further evidence from non-mammalian glial systems indicates that additional molecular mechanisms contribute to the dynamic control of mitochondria-lysosome contact sites in a context-dependent manner. For example, studies using Drosophila models have shown that the lysosomal SNARE (soluble N-ethylmaleimide-sensitive factor attachment protein receptor) proteins, including VAMP7 and VAMP8 (vesicle-associated membrane protein 7 and 8), regulate mitochondria-lysosome contact dynamics independently of canonical autophagic fusion. This regulation influences mitochondrial morphology, redox homeostasis, and lipid handling in glial cells [64]. These contacts are mechanistically distinct from mitophagic engulfment of damaged mitochondria, which is thought to be a classical function of the lysosome, highlighting a functional distinction between basal regulatory interactions and damage-induced degradative processes.

Notably, it is indicated that mitochondria-lysosomal crosstalk has a great impact on microglia activity as well as neuroinflammatory and neurodegenerative processes [65]. Despite the current lack of direct machinery evidence in microglia, insights from other systems provide a meaningful framework to study the principle of mitochondria-lysosome crosstalk. Taken together, accumulating evidence appears to support that microglial MCSs are lysosome- and immune-centered interaction hubs. Within these hubs, ER, mitochondria, and lysosomal compartments may be spatially coordinated to support rapid immune sensing and organelle remodeling. Both processes are accompanied by dynamic control of mitochondrial and lysosomal behavior and represent key architectural adaptations for future investigation.

Oligodendrocytes are highly specialized glial cells responsible for myelin sheath formation and maintenance, processes that impose extraordinary demands on lipid synthesis, membrane expansion, and spatial coordination of biosynthetic organelles. Despite their importance, MCSs in oligodendrocytes remain relatively underexplored compared with those in neurons and other glial cell types. Consistent with the lipid-intensive nature of myelination, available evidence suggests that MCSs in oligodendrocytes are largely centered around the endoplasmic reticulum (ER), particularly at ER–plasma membrane (PM) and ER–lipid droplet (LD) interfaces, which are known to support non-vesicular lipid transfer, lipid storage, and membrane expansion. During myelination, the ER extends into developing myelin sheaths and remains closely associated with expanding membrane layers, positioning ER-based lipid biosynthetic machinery near sites of membrane assembly. At the subcellular level, MCSs in oligodendrocytes are localized in the inner tongue of the myelin growing frontier, where tubular ER is enriched and distanced from the myelin membrane by gaps shorter than 20 nm. This meets the structural requirement for non-vesicular lipid transfer [66]. Notably, ER-myelin contact sites are development-dependent, highly enriched during the active phase of myelination at postnatal day 14 (P14) in mice, and are downregulated in mature myelin at 6 months of age, which is highly consistent with the spatiotemporal pattern of myelin growth [66].

Functionally, MCSs in oligodendrocytes fulfill three major roles. First, they mediate non-vesicular lipid transport to support myelin biosynthesis. ER-myelin membrane contact sites at the inner tongue region provide a functional platform for glycolipid transfer protein (GLTP). Through these sites, GLTP directly transfers ER-synthesized galactosylceramide (GalCer) to the myelin membrane, a process that represents a key pathway for myelin lipid accumulation. Conditional deletion of GLTP leads to reduced myelin glycolipid content, impaired myelination, and abnormal ER architecture [66]. Second, MCSs precisely regulate Ca^2+^ signaling and maintain Ca^2+^ homeostasis. ER-mitochondrial contact sites form Ca^2+^ release microdomains through enrichment of inositol 1,4,5-trisphosphate receptor 2 (IP3R2) and calreticulin. Mitochondria take up Ca^2+^ released from the ER to modulate local Ca^2+^ concentrations, thereby controlling the initiation, amplification, and propagation of Ca^2+^ waves. Inhibition of mitochondrial function markedly reduces Ca^2+^ wave propagation velocity and alters Ca^2+^ release kinetics [67,68]. In addition, these contact sites indirectly regulate IP_3_-mediated Ca^2+^ signaling by modulating the interaction between phosphatidylinositol 4,5-bisphosphate (PIP_2_) and gelsolin [67]. Third, oligodendrocyte MCSs participate in organelle coordination and stress defense. ER-mitochondria contact sites coordinate the metabolic activities of these two organelles through lipid exchange and Ca^2+^ signaling, contributing to oxidative stress responses and membrane stability. Dysfunction of these sites is closely associated with demyelinating disorders and neurodegenerative diseases [69].

In addition, ER-myelin contact sites represent a functionally specialized class of membrane contact sites in oligodendrocytes, reflecting the unique structural and metabolic demands of myelin biogenesis. Conceptually, these ER–myelin interfaces can be viewed as a specialized form of ER–PM contact sites, in which the myelin sheath constitutes a highly differentiated plasma membrane domain. These interfaces are thought to facilitate localized lipid transfer and membrane supply during myelin wrapping [66]. Ultrastructural and live-imaging studies have shown that myelin membrane growth occurs through the lateral expansion of compacted membrane sheets, a process that depends on continuous delivery of newly synthesized lipids from the ER [66]. Proteins enriched at ER-PM contacts, including VAP family members and lipid-transfer proteins [2], are therefore well positioned to support the spatial coupling between lipid synthesis and membrane accretion in oligodendrocytes.

Mature oligodendrocytes, in contrast to neurons, display relatively sparse mitochondrial content within compact myelin segments, and mitochondria are largely restricted to the cell body and non-compacted regions of the myelin sheath [69,70,71]. This spatial organization suggests that oligodendrocyte MCSs are less centered on ER-mitochondria Ca^2+^ signaling and instead emphasize ER-mediated lipid handling and membrane trafficking. Current knowledge is largely restricted to lipid- and myelin-related ER-centered architectures that are structurally and functionally adapted to sustain membrane production and long-term maintenance of axonal insulation, whereas other classes of oligodendrocyte MCSs remain poorly characterized.

## 3. Spatial Specialization of Membrane Contact Sites

**Figure 2:**
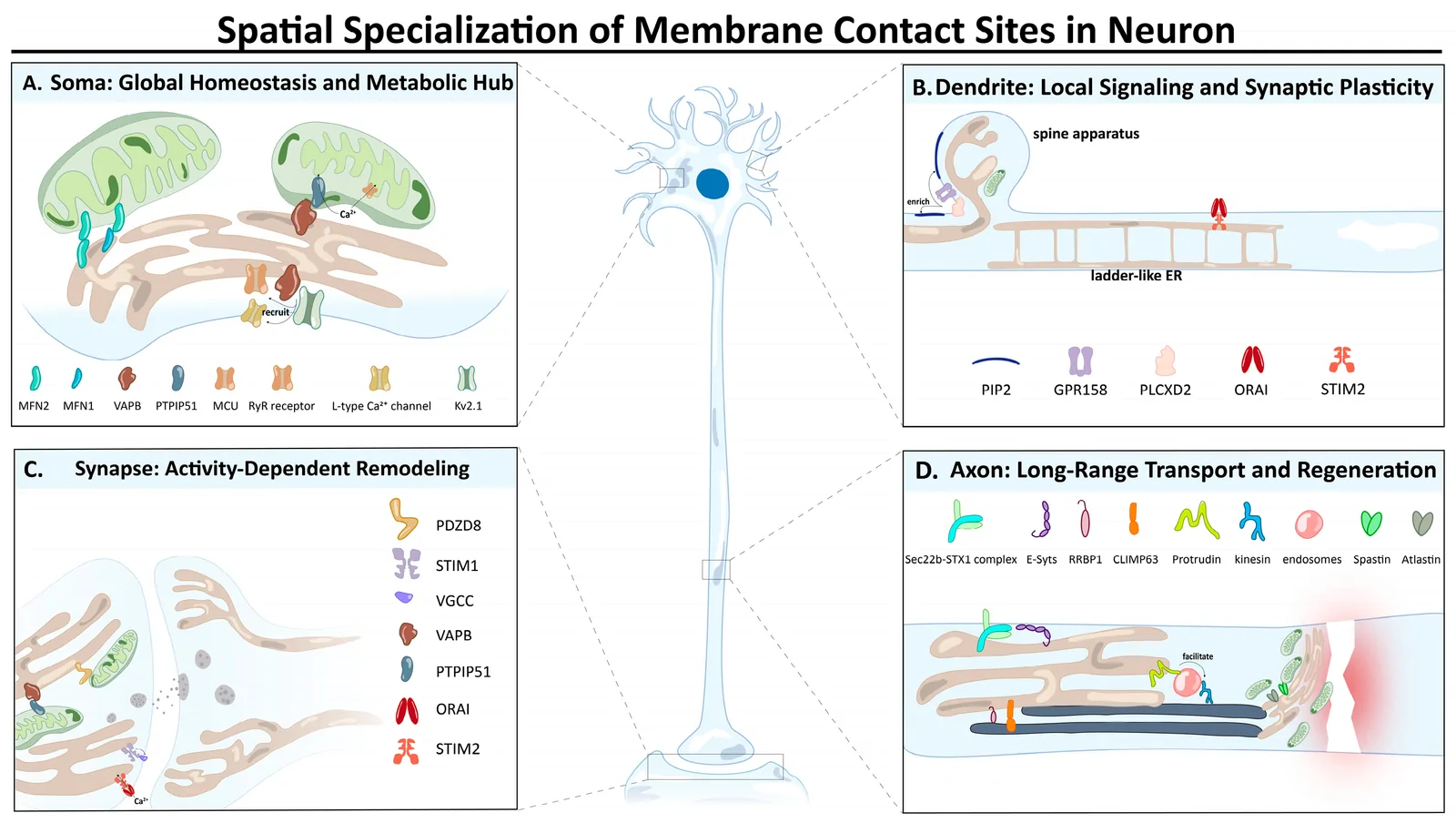
Spatial specialization of membrane contact sites in neurons. Neurons exhibit highly compartmentalized MCS organization aligned with local functional demands. (**A**). Somatic MCSs: Extensive ER cisternae form broad contacts with mitochondria and the plasma membrane (PM). ER-mitochondria contacts are stabilized by the VAPB-PTPIP51 and MFN1/2 tethering complexes, while ER-PM contacts are organized by Kv2.1-VAPB interactions and recruit L-type Ca^2+^ channels and RyRs to modulate membrane excitability. (**B**). Dendritic MCSs: ER-mitochondria and ER-PM contacts extend into spines. The spine apparatus is a specialized ER-PM contact stabilized by GPR158-PLCXD2 complexes (PIP2 enrichment by this complex is essential for spine maturation and stability). Ladder-like ER architecture (a periodic ER architecture that forms discrete ER-PM contact sites) involves STIM-ORAI complexes and enables long-range Ca^2+^ signal propagation. (**C**). Synaptic MCSs: Dynamic MCSs couple activity to Ca^2+^ signaling and ATP production. At presynaptic terminals, the VAPB-PTPIP51 complex stabilizes synaptic ER-mitochondria coupling; PDZD8 regulates mitochondrial Ca^2+^ uptake during activity, while STIM1-VGCC and STIM2-ORAI form a regulatory loop to fine-tune glutamate release probability. (**D**). Axonal MCSs: ER-PM and ER-endosome contacts facilitate lipid transfer, organelle transport, and membrane expansion, while injury-induced remodeling of ER-mitochondria contacts contributes to regeneration. Sec22b-ESYT2-STX1 facilitates non-vesicular lipid transfer for axonal outgrowth. Protrudin links the ER to late endosomes, recruiting Kinesin-1 motors for long-distance transport. RRBP1 and CLIMP63 link the ER to microtubules, coordinating ER motility and axonal transport, ensuring uniform ER distribution. Upon injury, Spastin and Atlastin remodel MCSs to promote ER accumulation at the injured sites. MAMs are enriched at regeneration zones.

Neurons exhibit an extreme degree of spatial compartmentalization, with distinct subcellular domains, including the soma, dendrites, axon initial segment, and synaptic terminals, performing highly specialized functions. This architectural complexity imposes unique constraints on organelle positioning, dynamics, and communication. Accordingly, membrane contact sites in neurons are not uniformly distributed but are spatially organized to meet local signaling and metabolic demands (Figure 2). This spatial specialization contrasts with glial cells, in which subcellular compartmentalization is less pronounced and the spatial organization of MCSs remains less well defined.

### 3.1. Somatic Versus Dendritic: Global Homeostasis Versus Local Signaling

In neurons, MCSs are distributed primarily in the soma and are dominated by large, flat ER cisternae closely apposed to other organelles, supporting global cellular homeostasis, including protein synthesis, lipid metabolism, and basal Ca^2+^ buffering. ER-mitochondria MCSs in the soma are stabilized by tethering complexes such as VAPB-PTPIP51 and MFN1/2-MFN2; these facilitate efficient Ca^2+^ transfer via IP_3_Rs and mitochondrial calcium uniporter (MCU), activating tricarboxylic acid (TCA) cycle dehydrogenases to support the high metabolic demands of the neuronal soma [27,32,72,73]. These contacts are critical for sustaining ATP production, which is required for somatic processes, including protein synthesis and ion gradient maintenance. In addition, ER-PM contact sites are especially abundant in the soma and often involve extensive subsurface cisternae with narrow luminal spacing, consistent with a role in sustained Ca^2+^ handling and lipid exchange [4]. Organized by voltage-gated potassium channel Kv2.1 (Kv2.1)-VAP interactions, these ER-PM contacts recruit L-type Ca^2+^ channels and ryanodine receptors (RyRs) to modulate membrane excitability through localized Ca^2+^ signaling, linking depolarization to intracellular responses [27,74]. The subsurface cisternae—specialized somatic ER-PM MCSs—further contribute to excitability regulation via Cav1-RyR2-KCa3.1 complexes, which require junctophilins for tethering and mediate after hyperpolarization [27,75].

In contrast, dendritic MCSs are more spatially restricted and specialized for local signaling and synaptic plasticity, with distinct roles in spine maturation and activity-dependent remodeling. The ER-mitochondria and ER-PM contacts are positioned along dendrites and extend partially into spines [4], though they are smaller than those in the soma. ER-PM contacts sometimes form a structure called the spine apparatus, which is stabilized by PIP_2_ enrichment via the G protein-coupled receptor GPR158-PLCXD2 (158-phosphatidylinositol-specific phospholipase C X domain-containing protein 2) complex, thereby supporting spine maturation and stability [76]. These MCSs facilitate local Ca^2+^ signaling through RyRs and IP_3_Rs, amplifying postsynaptic Ca^2+^ transients that drive long-term potentiation (LTP) [27,72]. ER-mitochondria MCSs in dendrites, mediated by VAP proteins, stabilize mitochondria at synapses to provide localized energy and modulate Ca^2+^ dynamics during plasticity. Furthermore, the periodic “ladder-like” ER architecture in dendrites creates discrete MCSs with the PM, enabling long-range Ca^2+^ signal propagation between synaptic inputs via STIM-ORAI (stromal interaction molecules-ORAI calcium release-activated calcium modulator) complexes [77,78,79].

### 3.2. Synaptic MCSs and Activity-Dependent Remodeling

MCSs form highly dynamic subcellular microdomains at the synaptic level, allowing direct coupling of neural activity to the membrane system and rapid regulation of Ca^2+^ signals. Both presynaptic and postsynaptic compartments contain specialized ER structures that form transient contacts with the plasma membrane, mitochondria, and endosomal compartments, which are much smaller than their somatic counterparts [4]. Recently, increasing attention has been directed toward MAMs at synapses, as these highly specialized compartments require tight coordination between Ca^2+^ signaling and local energy supply to sustain synaptic transmission. Studies have shown that MAMs at synapses can efficiently support ATP generation and maintain the synaptic vesicle cycle by delivering Ca^2+^ to mitochondria and promoting oxidative phosphorylation [80]. This is mainly regulated by the VAPB-PTPIP51 complex, which is abundant at synapses [37]. Deficiency in this complex can significantly inhibit synaptic activity and reduce the synaptic vesicle exocytosis [37].

Presynaptic ER-PM MCSs play dual roles in Ca^2+^ homeostasis: STIM2-ORAI complexes mediate store-operated Ca^2+^ entry (SOCE) to enhance spontaneous glutamate release, while STIM1 inhibits Voltage-Gated Calcium Channels (VGCCs) to reduce release probability, creating a fine-tuned regulatory loop [27,80]. ER-mitochondria MCSs in presynaptic terminals, dependent on ganglioside-induced differentiation-associated protein 1 (GDAP1) and PDZD8, support Ca^2+^ transfer for mitochondrial ATP production, which is critical for sustaining high-frequency synaptic vesicle release [27,72]. PDZD8 is also essential for mitochondrial Ca^2+^ uptake following synaptically evoked ER Ca^2+^ release, thereby helping to shape Ca^2+^ dynamics [41]. Activity-dependent remodeling of these MCSs is evident during LTP, in which extended synaptotagmin-1 (ESYT1)-mediated enhancement of ER-PM contact promotes α-amino-3-hydroxy-5-methyl-4-isoxazolepropionic acid (AMPA) receptor surface expression [81].

Postsynaptic MCSs are equally dynamic: ER entry into dendritic spines is activity-dependent and requires myosin Va and septin 3, whereas septin 3 functions to hold the ER in place after its delivery to the spine [82,83,84], thereby increasing the likelihood of stabilizing ER-PM contacts. During LTP induction, spine apparatus MCSs expand, increasing local Ca^2+^ storage and release capacity [72]. Conversely, synaptopodin loss—preceding spine elimination during pruning—disrupts spine apparatus MCSs, highlighting their dependence on synaptic input [85]. Additionally, postsynaptic ER-mitochondria MCSs are regulated by synaptic activity, with enhanced Ca^2+^ transfer supporting mitochondrial metabolism during prolonged synaptic activation [41].

### 3.3. Axonal and Long-Range MCSs: Supporting Transport and Resilience

Axonal MCSs are uniquely adapted to support the long-distance transport, membrane expansion, and resilience of axons, which often extend over millimeters to meters. Axonal ER-PM contact sites, which are typically smaller than those in the cell body [4], are critical for lipid transport and membrane growth. The vesicle-trafficking protein SEC22b (Secretory 22b)-ER membrane protein ESYT2 (extended synaptotagmin-2)-presynaptic PM protein STX1 (syntaxin-1) complex mediates non-vesicular lipid transfer, fueling axonal outgrowth [86], and further growth is supported by protrudin-mediated ER-late endosome MCSs. Protrudin, an ER-anchored protein, contains a PtdIns(3)P-binding Fab1, YOTB, Vac1, and EEA1 homology (FYVE) domain and interacts with the endosome through PtdIns(3)P [87]. These contact sites facilitate recruitment of the microtubule motor kinesin 1 to the late endosome and endosomal translocation to growth cones, thereby enabling membrane expansion during neurite outgrowth [88]. Axonal ER also interacts with microtubules through ribosome-binding protein 1 (RRBP1; also known as p180) and cytoskeleton-linking membrane protein of 63 kDa (CLIMP-63) to coordinate ER motility and its axonal transport, ensuring uniform ER distribution along the axon to support different cellular physiological functions [27,89]. However, whether this interaction constitutes an authentic membrane contact site remains to be defined.

Upon axotomy, ER accumulates at regeneration sites via spastin (SPAST) and atlastin (ATL)-dependent MCS remodeling [27], with both the ER and the mitochondria recruited and forming contacts at the injury sites. GRP75 is thought to be translated locally by injured axons and to promote axon regeneration, likely by enhancing Ca^2+^ transfer and ATP synthesis [90]. Long-range Ca^2+^ waves, which propagate via ER MCSs along the axon, can transmit the signal into the nucleus and trigger transcriptional responses to activate regeneration pathways [27]. Moreover, axonal ER-phagy (reticulophagy) machinery, mediated by family with sequence similarity 134 member B (FAM134B) and ATL3, regulates ER turnover, prevents pathological ER accumulation, and maintains axonal homeostasis [80].

Together, these findings demonstrate that neuronal MCSs are spatially patterned to support compartment-specific functions, ranging from global metabolic maintenance in the soma to rapid signaling and plasticity at synapses. Spatial specialization allows neurons to locally tune inter-organelle communication without perturbing whole-cell homeostasis. Importantly, disruption of this fine-grained organization, whether by genetic mutations, excitotoxic stress, or neurodegenerative disease, can selectively impair vulnerable compartments, such as synapses or axons, long before overt neuronal loss occurs.

Compared with neurons, glial cells exhibit less well-defined subcellular compartmentalization, and current studies provide limited information on the subcellular localization and compartment-specific organization of membrane contact sites in glial cells. A nanoscale reconstruction of reactive astrocytes has revealed subcompartment-specific organization of intracellular organelles, including MCSs between mitochondria and the ER. In particular, astrocytic endfeet (perivascular processes) exhibit a strikingly high abundance of ER-mitochondrial contact sites compared with non-endfoot regions [58,59], indicating the specialized metabolic requirements of these regions to support vascular remodeling.

## 4. Functional Consequences of Specialized MCSs

Spatial and cell-type-specific organization of MCSs is not merely a structural feature but directly shapes core cellular functions in the brain. In neurons and glial cells, MCS specialization determines how organelles coordinate Ca^2+^ signaling, metabolic fluxes, lipid and iron homeostasis, and stress responses. Disruption of this finely tuned organization, therefore, has profound consequences for neuronal excitability, synaptic integrity, and vulnerability to disease.

### 4.1. Ca^2+^ Signaling, Excitation-Metabolism Coupling, and Apoptosis

Ca^2+^ homeostasis and spatiotemporal dynamics represent a central regulatory hub in both neuronal and glial physiology, integrating membrane excitability with intracellular organelle signaling. In neurons, Ca^2+^ influx is tightly controlled through multiple pathways, including voltage-gated Ca^2+^ channels, N-methyl-D-aspartate (NMDA) receptors, Ca^2+^-permeable AMPA receptors, nicotinic acetylcholine receptors, purinergic P2X receptors, store-operated Ca^2+^ entry (SOCE), and transient receptor potential (TRP) channels, as well as through Ca^2+^ release from ER stores via IP_3_ and RyRs [91]. These pathways orchestrate neurotransmitter release, dendritic excitability, and activity-dependent synaptic plasticity [92]. Astrocytes, which are intimately associated with synapses, exhibit distinct Ca^2+^ signaling dynamics that are not strictly governed by neuronal action potentials. In astrocytes, Ca^2+^ signals are primarily generated by IP_3_-dependent ER release and manifest as oscillatory transients within fine processes that interface with synapses [93]. Mitochondrial positioning and ER-mitochondria coupling further shape Ca^2+^ dynamics, linking intracellular Ca^2+^ signaling to metabolic regulation and glial functional maturation.

Ca^2+^ signaling represents one of the most critical functional outputs of MCS, especially MAMs, in neural and glial cells. In neurons, ER-mitochondria contacts enable rapid Ca^2+^ transfer from the ER to mitochondria via the IP_3_R-GRP75-VDAC1 axis, functionally coupling synaptic activity to mitochondrial ATP production [32,94]. This spatial organization ensures that bursts of synaptic Ca^2+^ release are efficiently buffered and translated into enhanced metabolic responses, thereby sustaining neurotransmission under high energetic demand. This axis can respond to many Ca^2+^-related protein changes [95]. The functional importance of this coupling becomes evident when ER-mitochondria contacts are experimentally altered.

Beyond global excitation-metabolism coupling, spatial specialization of ER-mitochondria contacts refines Ca^2+^ signaling at dendritic and synaptic compartments. For example, PDZD8-dependent tethering in dendrites facilitates localized mitochondrial Ca^2+^ uptake following synaptically induced ER Ca^2+^ release, shaping cytosolic Ca^2+^ transients and influencing synaptic activity. Loss of PDZD8 prolongs cytosolic Ca^2+^ elevations without grossly altering organelle morphology, demonstrating that MCS specialization fine-tunes Ca^2+^ signaling with high spatial precision [41]. In addition to its tethering function, PDZD8 collaborates with protrudin and VAP proteins to regulate lipid trafficking, linking Ca^2+^ handling at MCSs to broader aspects of neuronal homeostasis [96,97,98].

The identification of molecular players involved in MCS regulation has been a major focus of research aimed at understanding how MCS dynamics are modulated. The Sigma-1 receptor (S1R), a non-opioid ER membrane protein [99] chaperone, is enriched at MAMs, is widely expressed in the nervous system, and stabilizes IP_3_R3 and sustains ER-to-mitochondria Ca^2+^ flux under physiological conditions [100]. Upon Ca^2+^ depletion or pharmacological activation, S1R dissociates from 78 kDa glucose-regulated protein (GRP78; also known as binding immunoglobulin protein, BiP) and redistributes within ER membranes [101], enabling dynamic modulation of Ca^2+^ signaling and inter-organelle communication. These properties position S1R as a fine tuner rather than a primary determinant of Ca^2+^ flux at MCSs.

Another protein implicated in shaping Ca^2+^ dynamics at ER-mitochondria contacts is Reticulon-1A (RTN1A), a neuron-enriched ER protein localized to smooth ER in dendrites and known to interact with RyR2, an ER Ca^2+^ release channel [102,103]. Quantitative proteomic and functional analyses identified RTN1A as a prominent ER-mitochondrial contact-associated protein that markedly promotes ER-mitochondrial contacts [104]. The N-terminal domain of RTN1A is essential for this activity, as its deletion abolished ER-mitochondria contact formation, while fusion of the RTN1A N-terminus to another reticulon restored function [104]. While these findings suggest a potential role for RTN1A in neuronal Ca^2+^ homeostasis, its physiological roles, particularly in the context of organelle communication and neuronal function, remain incompletely understood, highlighting RTN1A as an intriguing and understudied regulator of membrane contact sites in the nervous system.

Similarly, components of ER membrane quality-control pathways, such as GET4 (guided entry of tail-anchored proteins factor 4) and BAG6 (BCL2-associated athanogene) have been reported to influence the abundance of ER-mitochondrial contacts and mitochondrial Ca^2+^ uptake in specific experimental systems. Studies have found that GET4 and BAG6, which can interact through heat shock protein 70 (HSP70) [105] and participate as a protein complex in ER membrane quality control by sorting proteins that fail to insert properly into the membrane, can interact separately with either IP3R or GRP75 to modulate Ca^2+^ dynamics in mitochondria [106]. In a Drosophila model of familial AD, neuronal suppression of GET4 increases ER-mitochondrial contact sites and robustly rescues multiple Aβ42-Arc-associated neurodegenerative phenotypes, indicating that enhancing ER-mitochondrial contacts can exert neuroprotective effects in vivo [106]. However, the direct relevance of these pathways to neuronal Ca^2+^ signaling remains unclear, and their contribution to MCS function is likely to be highly context dependent.

The physiological importance of ER–mitochondria Ca^2+^ coupling becomes particularly evident under pathological conditions. Consistent with the context-dependent role of Mfn2 discussed above, Mfn2 knockdown has been reported to enhance ER–mitochondria coupling and subsequently to increase susceptibility to Ca^2+^-dependent cell death in some experimental systems. This is because increased ER–mitochondria crosstalk promotes mitochondrial Ca^2+^ uptake, leading to mitochondrial Ca^2+^ overload and ultimately rendering cells more vulnerable to cell death [20,47]. Under physiological conditions, ER–mitochondria Ca^2+^ transfer at MAMs is tightly regulated to meet cellular demands for mitochondrial ATP production. However, sustained or excessive Ca^2+^ flux through the IP3R–GRP75–VDAC1 axis, followed by uptake through MCU, results in mitochondrial Ca^2+^ overload [94,107]. Elevated matrix Ca^2+^ not only stimulates excessive oxidative phosphorylation and reactive oxygen species (ROS) production but also sensitizes mitochondria to permeability transition, leading to dissipation of the mitochondrial membrane potential (ΔΨm) and increased outer mitochondrial membrane permeabilization [108,109]. Consequently, cytochrome c is released into the cytosol, where it binds apoptotic protease activating factor-1 (APAF-1) in the presence of ATP/dATP to assemble the apoptosome [109,110]. The apoptosome subsequently initiates the intrinsic caspase cascade through activation of executioner caspases, ultimately leading to apoptotic cell death. Therefore, while physiological ER–mitochondria Ca^2+^ coupling is indispensable for metabolic homeostasis, dysregulated MAM-mediated Ca^2+^ transfer provides a direct mechanistic link between organelle contact site dysfunction and neuronal loss in neurodegenerative diseases.

More recent studies have shown that the physical parameters of membrane contact sites may influence their function in regulating Ca^2+^ metabolism. An experiment using HeLa cells employed molecular spacers of different lengths to stabilize the MAM intermembrane distance at different ranges (5, 10, 15, 20, and 30 nm) [111]. Among all the distances tested, a 20 nm spacing was found to be the most favorable for IP3R enrichment and mitochondrial calcium uptake [111]. This finding was also validated in a mouse AD astrocyte model and in human Parkinson’s Disease (PD) iPSC-derived astrocytes. In the former, stabilizing MAM at 20 nm with a linker restored mitochondrial Ca^2+^ uptake and protein quality control, whereas a 10 nm linker did not [112]. In the latter, PD cells exhibited a significant reduction in 20 nm MAM contacts accompanied by decreased mitochondrial Ca^2+^ uptake; restoring 20 nm, but not 10 nm, contacts rescued this defect [111]. These findings may support a very interesting point: in neural cells, shorter membrane contacts are not necessarily more effective; instead, specific functions appear to have an optimal distance window.

In summary, these findings establish Ca^2+^ transfer at membrane contact sites as a central functional consequence of MCS specialization, with ER-mitochondria coupling acting as a tunable platform that balances metabolic adaptation, plasticity, and vulnerability across neuronal and glial cell types.

### 4.2. Lipid, Iron, and Metabolic Homeostasis

MCSs constitute essential platforms for non-vesicular lipid synthesis, modification, and inter-organelle transport in the nervous system, where precise control of membrane composition is critical for neuronal signaling and glial support functions. In neurons, the ER, as the principal site of lipid biosynthesis, establishes extensive contacts with multiple organelles throughout the neuron, providing an anatomical basis for local lipid exchange independent of long-range vesicular trafficking [4].

Among these interfaces, MAMs play a central role in phospholipid metabolism. At MAMs, phosphatidylserine (PS) synthesized in the ER is transferred to mitochondria and converted into phosphatidylethanolamine (PE), a lipid essential for mitochondrial membrane integrity and respiratory function. Impairment of ER-to-mitochondria PS transfer compromises PE synthesis and is sufficient to induce mitochondrial dysfunction, highlighting MCS architecture as a critical constraint on mitochondrial lipid homeostasis and bioenergetic capacity, particularly in neurons with high metabolic demand [113].

In addition to ER-mitochondrial interfaces, ER-PM contact sites are major regulators of lipid homeostasis in neurons. The brain-enriched lipid transporter TMEM24 (transmembrane protein 24; formerly known as C2 Ca^2+^-dependent domain-containing protein 2-like, C2CD2L) localizes to ER-PM junctions and mediates phospholipid transfer from the ER to the plasma membrane. TMEM24 dynamically dissociates from ER-PM contacts in response to cytosolic Ca^2+^ elevations, providing an activity-dependent mechanism that couples neuronal excitation to rapid remodeling of plasma membrane lipid composition [114]. More recent work has demonstrated that TMEM24 and its paralog C2CD2 are preferentially enriched at ER-PM junctions formed at cell–cell contact sites, suggesting that lipid transfer at neuronal and neuron-glia interfaces may support localized signaling and adhesion domains [115].

In glial cells, MCS-mediated lipid metabolism is tuned toward lipid storage, buffering, and membrane expansion, consistent with their metabolic support functions. Quantitative imaging approaches reveal that neurons and astrocytes exhibit distinct organelle signatures and inter-organelle interaction patterns, supporting the concept of cell-type-specific MCS specialization [16]. In astrocytes, ER-LD contacts promote LD biogenesis and neutral lipid storage, while mitochondria-LD contacts regulate fatty-acid utilization and oxidative metabolism. Dysregulation of these lipid-handling contact sites in glia can indirectly compromise neuronal function by altering the lipid environment required for synaptic and axonal integrity [23].

Mechanistically, several bridge-like lipid transport proteins explain how MCS organization directly enforces lipid-metabolic wiring. Members of the vacuolar protein sorting-associated protein 13 (VPS13) family possess extended hydrophobic grooves that facilitate bulk, non-vesicular lipid transfer between membranes. VPS13A and VPS13D localize to ER-mitochondrial contact sites and regulate mitochondrial morphology and function, whereas other family members localize to distinct ER-organelle interfaces [116]. VPS13A displays differential expression across neuronal subtypes and is detected only in selected glial populations, suggesting that cell-type-specific expression of VPS13 proteins may underlie selective vulnerability in neurodegenerative disease [117]. Loss of VPS13 function in neurons results in elongated, dysfunctional mitochondria with altered membrane potential, further linking lipid transfer defects to mitochondrial pathology [118]. In addition to lipid transporters, regulatory proteins shape local lipid microdomains at MCSs. Accumulating evidence supports a model in which the S1R acts as an organizer of cholesterol-enriched microdomains at the ER, preferentially at MAMs [101]. These domains include distinct lipid and protein compositions and increased membrane thickness, and are proposed to provide specialized platforms for protein post-translational modification, maturation, and early folding events prior to trafficking to post-ER organelles [101]. Notably, such lipid raft-like ER subdomains are upregulated in AD, further implicating MCS-associated lipid organization in neurodegenerative pathology [119].

Mitochondria and lysosomes are key components of cellular iron homeostasis. Mitochondria serve as the exclusive site of heme biosynthesis and a major platform for iron-sulfur cluster (ISC) assembly, making them central to cellular iron metabolism. Efficient mitochondrial iron utilization requires close coordination with cytosolic and endolysosomal iron pools. Studies on mitochondrial iron trafficking have established a framework for understanding how mitochondrial iron utilization is coordinated with extra-mitochondrial iron metabolism, providing a conceptual basis for the potential importance of proximity-based organelle crosstalk [120]. On the other hand, lysosomes act as major intracellular iron reservoirs by degrading iron-containing macromolecules and organelles, thereby releasing redox-active iron that must be safely redistributed or reutilized [121]. Emerging evidence from cancer cell systems suggests that mitochondria-lysosome contact sites directly participate in intracellular iron trafficking via 3-hydroxybutyrate dehydrogenase 2 (BDH2) [122]. However, whether MCSs between these two organelles are directly involved in iron transfer in the nervous system remains unknown. It is worth noting that in neurodegeneration with brain iron accumulation (NBIA), excessive iron deposition occurs alongside mitochondrial and lysosomal dysfunction, suggesting a potential link between impaired mitochondrial-lysosome contacts and dysregulated iron trafficking [123].

Ferroptosis is an iron-dependent form of regulated cell death characterized by the peroxidation of membrane phospholipids [124]. MAMs have emerged as critical hubs for integrating multiple signals involved in ferroptosis. For example, studies have shown that following ferroptosis induction, phospholipid peroxides first aggregate at these contact sites and subsequently propagate toward mitochondria via expansion of these contacts [125]. This process is accompanied by mitochondrial ROS generation, fragmentation, and further amplification of cellular lipid peroxidation [125]. A complementary mechanism for ferroptosis seems to involve MAM-dependent Ca^2+^ and ROS signaling [126]. Pharmacological targeting of the MAM-resident chaperone S1R, or genetic disruption of MAM integrity, impaired ER-to-mitochondria Ca^2+^ transfer, altered mitochondrial ROS production, and caused accumulation of polyunsaturated fatty acid (PUFA)-containing triacylglycerols. These findings suggest that functional MAMs promote ferroptosis not only by facilitating mitochondrial oxidative signaling but also by influencing whether PUFAs remain stored in neutral lipids or become available for incorporation into oxidation-sensitive membrane phospholipids [126]. Moreover, studies have shown that ER–mitochondria contacts are not uniformly pro-ferroptotic but also have the ability to organize local protective signaling in part via the AKAP1 (A-kinase anchoring protein 1)-PKA (protein kinase A)-GRP75 pathway in a colon cancer model [127]. Taken together, these studies indicate that MAMs can simultaneously control reactions both driving and opposing ferroptosis.

Additional lines of evidence linking MAMs and ferroptosis have emerged from multiple disease model systems. In a spinal cord injury model, neuronal ferroptosis is linked to excessive expansion of MAMs as well as increased mitochondrial lipid peroxidation [128]. In a PD model, current evidence supports a bidirectional pathological network in which mitochondrial respiratory defects and ROS production enhance lipid peroxidation, while altered membrane lipids promote α-synuclein aggregation, impair mitophagy, and further destabilize mitochondria. Disrupted ER–mitochondria communication contributes to this network by altering phospholipid trafficking, mitochondrial membrane composition, Ca^2+^ handling, and oxidative stress, thereby increasing dopaminergic-neuron susceptibility to ferroptosis [129]. Another example comes from a study of epilepsy [130]. MAMs were shown to integrate Ca^2+^ signaling, ROS, iron, PUFA and antioxidative system in a nanoscale space, making them a catalytic platform for ferroptosis. MAM-driven ferroptosis (via redox signaling) was proposed as a key potential mechanism underlying epilepsy; the chronic progression of epilepsy can be viewed as a self-perpetuating pathological cycle in which MAM remodeling promotes ferroptosis, leading to neuronal loss, neuroinflammation, and ultimately maladaptive network reorganization [130]. However, these disease-specific relationships are currently based largely on the integration of parallel abnormalities rather than on direct demonstration that a defined MCS complex initiates ferroptosis in disease neurons. Nevertheless, these findings support a model in which iron overload is not only a metabolic insult but also provokes a maladaptive reconfiguration of organelle communication, including contact-dependent pathways that converge on mitochondrial fragmentation and loss of neuronal viability.

Beyond iron, MCSs also support metabolically consequential organization of intracellular logistics in polarized neurons. Notably, late endosomes can serve as platforms for local mRNA translation in axons and thereby sustain mitochondrial function and axonal integrity, as they form dynamic contact sites with mitochondria that serve as translational hotspots and enable the local synthesis and immediate supply of proteins required for mitochondrial function and maintenance in distal axons [131]. This illustrates how endolysosomal compartments can participate in spatially restricted metabolic support programs that are especially relevant for long neuronal projections.

Although MCSs provide a shared structural framework for iron and metabolite coordination in both neurons and glia, the physiological emphasis of these networks is likely cell-type specific. Neurons may be particularly sensitive to iron-driven perturbations because iron imbalance can rapidly destabilize mitochondrial function and redox homeostasis, whereas glial cells, especially astrocytes, are generally equipped with robust endolysosomal recycling capacity that can buffer extracellular and intracellular metabolic fluctuations. This cell-type specificity reflects an emerging consensus in the field that the functional roles of brain MCSs vary with cellular context, particularly influencing mechanisms involved in aging and neurodegenerative processes [25]. Furthermore, it provides a useful conceptual lens for understanding why the breakdown of endolysosomal-mitochondrial coordination can translate into neuron-selective vulnerability, even when glial compartments are heavily engaged in recycling and metabolic support.

### 4.3. Stress Responses, Quality Control, and Cell Vulnerability

MCSs also function as dynamic hubs that coordinate stress responses, organelle quality control, and cellular vulnerability in the brain. These contacts integrate Ca^2+^ signaling, mitochondrial metabolism, inflammatory pathways, and organelle turnover, thereby shaping how neurons and glial cells respond to physiological and pathological stress.

A central quality-control pathway linked to mitochondrial dysfunction is the PTEN-induced kinase 1 (PINK1)–Parkin system. In neurons, PINK1 localizes to MAMs and becomes further enriched upon mitochondrial depolarization induced by carbonyl cyanide m-chlorophenyl hydrazone (CCCP) [132]. Upon mitochondrial depolarization, PINK1 is selectively stabilized on the outer membrane of impaired mitochondria, where it accumulates due to the inhibition of voltage-dependent proteolysis. This accumulation is both necessary and sufficient to recruit cytosolic Parkin, which in turn promotes the autophagic elimination of damaged mitochondria. Disease-causing mutations in either PINK1 or Parkin disrupt distinct steps of this pathway, resulting in defective mitophagy and impaired mitochondrial quality control, mechanisms directly implicated in the vulnerability of dopaminergic neurons in Parkinsonism [133].

Consistent with this MAM-centered regulatory framework, members of the VPS13 family have emerged as important modulators of mitochondrial quality control at membrane contact sites. As mentioned above, VPS13 proteins are widely recognized as lipid transfer factors that function at inter-organelle interfaces, including ER–mitochondria contacts, with VPS13A and VPS13D particularly implicated in MAM-associated processes [134]. Notably, VPS13D has been shown to regulate mitophagy in a PINK1-dependent manner [135], suggesting that MCSs provide an additional layer of control over mitochondrial quality surveillance. In addition, although VPS13B is primarily localized to the Golgi apparatus and endosomal compartments [134], recent evidence indicates that it is recruited to DRP1-associated mitochondrial fission sites at MFN2-positive contact sites, regions where it promotes the delivery of phosphatidylinositol 4-phosphate (PI4P)-enriched Golgi-derived vesicles [118]. This spatially coordinated activity suggests that VPS13B functions at multi-organelle contact interfaces to facilitate late-stage membrane remodeling downstream of DRP1-mediated constriction.

Beyond its established role in regulating ER–mitochondria Ca^2+^ transfer (Section 2.1), S1R also coordinates adaptive ER stress signaling. Following ER stress, S1R dissociates from GRP78, stabilizes activated IP_3_R3 to preserve mitochondrial bioenergetics, and simultaneously stabilizes inositol-requiring enzyme 1 (IRE1) at MAMs, facilitating IRE1-X-box binding protein 1 (XBP1) signaling and promoting cell survival. Disruption of S1R-dependent signaling compromises both ER stress adaptation and mitochondrial function and has been associated with multiple neurodegenerative diseases [97,120,121].

ER-mitochondria contacts are increasingly recognized as organizers of inflammatory stress responses [136,137]. MAMs provide a molecular platform essential for the assembly and activation of the NLRP3 inflammasome, supporting the recruitment of inflammasome components and the maturation of pro-inflammatory cytokines such as interleukin-1β [138]. The adaptor protein mitochondrial antiviral-signaling protein (MAVS) is required for mitochondrial localization and activation of NLRP3, linking antiviral and inflammatory signaling to ER-mitochondria interfaces. Importantly, several proteins associated with neurodegenerative diseases, including α-synuclein and presenilins, localize to MAMs and/or influence their integrity, and their dysregulation is associated with altered ER-mitochondria architecture and exaggerated inflammatory signaling [139].

The integrity of ER morphology itself also contributes to stress sensitivity. Protrudin, an ER-shaping protein mutated in a subset of hereditary spastic paraplegia cases, localizes predominantly to tubular ER and promotes ER network formation and stabilization [140]. Pathogenic protrudin mutants exhibit increased intracellular stability and induce heightened susceptibility to ER stress, highlighting how altered ER structure can indirectly compromise ER-mitochondria functional coupling and cellular stress tolerance in neurons [141].

Collectively, these studies support a model in which ER-mitochondria contact sites operate as central stress-response hubs in the brain, integrating mitochondrial quality control, ER stress signaling, inflammatory pathways, and organelle crosstalk. While adaptive remodeling of these contacts can promote survival, their genetic or pathological dysregulation shifts the balance toward impaired quality control and heightened cell vulnerability in both neurons and glial cells.

## 5. Disease-Driven Remodeling of Membrane Contact Sites in the Brain

Neurological diseases do not merely reflect a breakdown of normal MCS functions but actively reshape inter-organelle communication in a disease- and cell type-specific manner. Chronic pathological conditions such as neurodegeneration, excitotoxic injury, and neuroinflammation impose sustained metabolic and signaling stress that preferentially targets specific classes of MCSs and their proteins (Table 1), thereby amplifying pre-existing differences between neurons and glial cells.

### 5.1. Neurodegenerative Diseases

A growing body of evidence identifies MAMs as central pathological hubs in AD, where amyloid processing, mitochondrial dysfunction, lipid metabolism, and inflammatory signaling converge. Rather than representing a uniform structural abnormality, MAM remodeling in AD manifests as altered organelle coupling and redistribution of tethering proteins and lipid microdomains. These alterations can influence Ca^2+^ signaling, phospholipid metabolism, mitochondrial bioenergetics, and organelle quality control, thereby contributing to the pathology and molecular phenotypes mentioned above [17,18,19,20,22]. Both familial and sporadic AD models exhibit early remodeling of ER-mitochondria contact sites, supporting the idea that MAM dysregulation is not merely a consequence of neurodegeneration but an active contributor to disease progression [147,148,158,159].

Recently, MAMs have been shown to recruit amyloid precursor protein (APP), positioning them as major intracellular platforms for β-secretase-mediated amyloid-β (Aβ) generation [148]. The molecular mechanism responsible for this active recruitment remains incompletely resolved, yet evidence shows that APP undergoes palmitoylation in the ER, and the palmitoylated fraction (palAPP) is highly enriched in MAMs compared with non-palmitoylated APP, suggesting a role for palmitoylation in APP recruitment to MAMs [147]. Following β-secretase cleavage, increasing evidence indicates that the C-terminal fragment C99 preferentially accumulates in MAMs, where it serves as the substrate for MAM-associated γ-secretase. Importantly, C99 accumulation itself perturbs MAM lipid composition, increases sphingolipid turnover, disrupts mitochondrial respiratory super complexes, and impairs mitochondrial bioenergetics, suggesting that C99 is not merely an intermediate of Aβ production but also an active pathogenic species within MAMs [160,161,162]. Biochemical fractionation studies further demonstrate that β-site APP-cleaving enzyme 1 (BACE1) and γ-secretase components are present in MAMs [160]. These findings suggest that prior enrichment of molecular machinery responsible for APP processing at MAMs may also provide a potential mechanism for APP recruitment and accumulation at these sites. In contrast, although Aβ and amyloid intracellular domain (AICD) are generated by γ-secretase activity associated with MAMs [160,162], there is currently no convincing evidence that they are subsequently retained at or actively recruited back to MAMs. Likewise, no studies have demonstrated specific MAM recruitment of the soluble ectodomains sAPPα or sAPPβ.

Importantly, modulation of MAM abundance, particularly via the MAM-resident S1R, selectively alters Aβ production and release in neuronal processes and axons, highlighting a spatially restricted role of MAMs in pathogenic Aβ signaling [147,148,149]. In parallel, Aβ pathology feeds back onto MAM architecture by reinforcing MAM coupling. In Amyloid-β oligomers (Aβo)-treated hippocampal-derived cells, the expression of IP3R, S1R, GRP75, and VDAC1 was increased, together with selected MAM-associated proteins such as VAPB and MFN2. These changes are consistent with reinforcement of the IP3R–GRP75–VDAC1 pathway, which transfers Ca^2+^ released from the ER toward mitochondrial VDAC1 [143]. Consistently, Aβo enhances mitochondrial Ca^2+^ uptake following ER release, and inhibition of MCU increases Ca^2+^ transfer between these two organelles [143]. Emerging evidence further suggests that this remodeling is, at least in part, mediated by histone deacetylase 2 and 3 (HDAC2/3)-dependent transcriptional regulation of MAM-associated proteins [163]. In complementary AD models, increased ER-mitochondria connectivity was associated with altered mitochondrial membrane potential, oxygen consumption, ATP production, and autophagosome formation, indicating that Aβ-induced MAM remodeling has broader consequences for mitochondrial metabolism and cellular quality control [158]. Such changes enhance ER-mitochondria coupling and perturb mitochondrial Ca^2+^ handling, ATP production, and autophagy, forming a self-reinforcing pathological loop [158,159,164].

Beyond molecular remodeling, the physical spacing of membrane contact sites also critically influences their functional output. Using a 3D neural model, ER–mitochondria contacts were experimentally constrained to different lengths [148]. Contacts stabilized at 6 nm promoted Aβ production and reduced the axonal motility of MAM-associated mitochondria, whereas the physiological spacing of approximately 25 nm maintained baseline function. By contrast, increasing the intermembrane distance to approximately 40 nm suppressed Aβ generation but also significantly reduced the axonal distribution of MAM-associated mitochondrial structures [148].

Also, disruption of lipid metabolism at neuronal MCSs has emerged as a prominent feature of AD. Enhanced ER-mitochondria coupling is accompanied by abnormal lipid remodeling at MAMs. Accumulation of the APP-derived C99 fragment at MAMs leads to increased sphingolipid turnover and ceramide production, altering the lipid composition of both MAMs and mitochondrial membranes and impairing the assembly and activity of mitochondrial respiratory super complexes [161]. In parallel, MAMs exhibit lipid raft-like properties enriched in cholesterol and glycosphingolipids, and MAM function is upregulated in AD, providing a mechanistic framework linking altered lipid microdomains to amyloidogenic processing and mitochondrial stress [119].

Beyond amyloidogenesis, MAM remodeling in AD seems to reshape innate immune signaling as well. Following mitochondrial stress, NLRP3 and ASC relocate from the ER to the MAMs, where the ROS serves as a signal for inflammasome activation [21]. Aβ-dependent activation of the NLRP3 inflammasome in microglia relies on mitochondrial stress signals, including reactive oxygen species, which are spatially organized at ER-mitochondria interfaces [165,166]. Together, these findings support the “MAM hypothesis” of AD, proposing that pathological rewiring of membrane contact sites amplifies amyloid burden, mitochondrial stress, and inflammation, ultimately driving synaptic failure and neuronal loss.

PD has also been associated with abnormal MCSs, particularly ER-mitochondria and mitochondria-lysosome contact sites, mainly due to their functions in Ca^2+^ signaling, lipid transport, and mitochondrial quality control [18,19,167]. Genetic forms of PD caused by mutations in VPS13C, PINK1, Parkin, and related genes converge on MCS dysfunction, indicating that altered inter-organelle communication is a core pathogenic mechanism rather than a secondary consequence of neurodegeneration.

In neurons, PD-associated mutations primarily disrupt ER-mitochondria coupling, thereby compromising lipid exchange, Ca^2+^ handling, organelle communication, and mitochondrial maintenance. Loss of VPS13C, a lipid transfer protein at ER-endolysosome and ER-lipid droplet contacts, impairs intracellular lipid trafficking and indirectly compromises mitochondrial function, contributing to early-onset PD [116]. Neurons derived from PINK1- and Parkin-linked PD exhibit increased baseline MAM abundance but fail to dynamically remodel narrow contacts in response to Ca^2+^ stress, resulting in maladaptive Ca^2+^ distribution at contact sites and impaired mitochondrial uptake [167]. In parallel, α-synuclein disrupts MAM integrity by interfering with the VAPB-PTPIP51 tether and by forming membrane-permeabilizing oligomers, thereby linking MCS dysfunction to mitochondrial fragmentation, ATP depletion, and synaptic failure [18,166].

In glial cells, MCS dysregulation prominently affects lipid handling, vesicle trafficking, and non-cell-autonomous neurotoxicity. In PINK1-deficient models, disrupted Rab7-dependent trafficking in ensheathing glia alters organelle contact dynamics and lipid homeostasis, driving dopaminergic synapse loss independently of neuronal PINK1 status [156]. Additional PD-linked mutations further underscore glial vulnerability at MCSs: loss of Ca^2+^-independent phospholipase A2 group VIA (iPLA2-VIA; encoded by PLA2G6) alters membrane lipid composition and destabilizes MCS architecture, promoting ER stress and α-synuclein aggregation, while Ras homolog family member T1 (RHOT1; encoding mitochondrial Rho GTPase 1, Miro1) mutations reduce ER-mitochondria contacts and impair Ca^2+^ buffering and mitophagy, exacerbating mitochondrial loss [168,169]. Taken together, these findings indicate that PD pathogenesis involves cell-type-specific modes of MCS failure: neuronal MCS dysfunction primarily impairs Ca^2+^ coupling and mitochondrial bioenergetics, whereas glial MCS defects preferentially disrupt lipid metabolism and organelle trafficking. The convergence of these neuron- and glia-specific vulnerabilities at membrane contact sites provides a unifying framework for understanding selective dopaminergic neuron degeneration in PD [19,156,167,168,170,171].

Dysregulated iron homeostasis and abnormal iron accumulation have also been shown to influence PD progression [129,172]. Increased levels of labile Fe^2+^ promote the peroxidation of PUFA-containing phospholipids through Fenton chemistry, while damaged mitochondria continuously generate ROS and compromise ATP production and antioxidant capacity [129]. Impaired PINK1/Parkin-mediated mitophagy prevents the efficient clearance of these dysfunctional mitochondria [129,172], and weakening of the glutathione-GPX4 and nuclear factor erythroid 2-related factor 2 (Nrf2) defense systems allows lipid peroxides to accumulate [129]. In parallel, α-synuclein further disrupts membrane lipid homeostasis, mitochondrial function, and cellular clearance pathways, ultimately increasing the vulnerability of substantia nigra dopaminergic neurons to both ferroptotic and non-ferroptotic cell death [129]. Together, these findings suggest that iron accumulation in PD is not an independent event but forms a pathological network together with failed mitochondria quality control, dysregulation of membrane lipid homeostasis, a-synuclein accumulation, antioxidative defense failure, and MAM dysfunction.

The quantitative properties of MCSs are also altered in PD, although their causal contribution to disease pathogenesis remains incompletely understood. Live-cell imaging of human iPSC-derived midbrain dopaminergic neurons demonstrated that mitochondria–lysosome contacts dynamically form and dissociate throughout the soma, axons, and dendrites [157]. In neurons derived from patients with glucosylceramidase beta 1 (GBA1)-associated PD, contact duration is significantly prolonged, likely owing to impaired TBC1D15-mediated Rab7-GTP hydrolysis and defective untethering following reduced glucocerebrosidase (GCase) activity. Restoration of GCase activity or overexpression of TBC1D15 shortens contact duration and improves mitochondrial distribution, ATP production, and mitochondrial function [157].

MCS dysfunction also represents a converging pathogenic mechanism in hereditary spastic paraplegia (HSP). Across genetically diverse HSP subtypes, impaired ER-centered MCS organization links disrupted organelle homeostasis to selective degeneration of long corticospinal axons [173,174]. At ER-mitochondria interfaces, HSP-associated proteins directly regulate mitochondrial integrity and axonal maintenance [175]. ADP-ribosylation factor-like 6 interacting protein 1 (ARL6IP1), a MAM-localized protein, coordinates [97] mitophagy and ER-mitochondria coupling through interactions with microtubule-associated proteins 1A/1B light chain 3B (LC3B) and BCL2-like protein 13 (BCL2L13); its loss reduces ER-mitochondria contacts and induces mitochondrial dysfunction, axonal demyelination, and neuroinflammation [176]. Similarly, FIC domain protein adenylyltransferase (FICD) mutations disrupt ER proteostasis by dysregulating GRP78 AMPylation, leading to ER stress, reactive oxygen species accumulation, and motor impairment, thereby recapitulating key features of early-onset HSP, as demonstrated in a Drosophila HSP model [177]. In addition, classical HSP proteins involved in ER shaping, including atlastin-1 (SPG3A), spastin (SPG4), reticulon (SPG12) and receptor expression-enhancing protein 1 (REEP1; SGP31), are required for proper MCS architecture, and their mutations impair axonal transport and mitochondrial function [104,152,153,178]. Specifically, REEP1 functions at the ER–mitochondria interface to regulate inter-organelle coupling, and HSP-associated mutations disrupt this interaction, contributing to neuritic growth defects and degeneration [152].

Beyond ER-mitochondria contacts, ER-endosome MCS dysfunction contributes to axonal pathology in HSP by disrupting lipid transfer and endosomal maturation. Protrudin (SPG33) forms a complex with PDZD8 at ER-endosome contact sites to support neuronal polarity, and pathogenic mutations lead to abnormal enlargement of late endosomes and axonal degeneration [97]. Spastin-dependent ER-endosome contacts further regulate endosomal fission and lysosomal trafficking through interactions with endosomal sorting complexes required for transport (ESCRT)-III components, and disruption of this pathway results in lysosomal dysfunction and axonal pathology [179,180]. Importantly, MCS dysfunction in HSP is not neuron-restricted: SPG3A mutations perturb astrocytic lipid droplet dynamics and cholesterol transfer to neurons, contributing to neuronal axonal degeneration [175,179]. More broadly, dysregulated MCS signaling promotes proinflammatory microglial activation and neuronal inflammation, together exacerbating axonal degeneration [23].

Consistent with the above-mentioned neurodegenerative diseases, studies of amyotrophic lateral sclerosis/frontotemporal dementia (ALS/FTD) have increasingly converged on the idea that contact-site failure is not simply secondary to neurodegeneration, but may act as an upstream driver that coordinates multiple pathological phenotypes [181,182,183]. For example, oxidative stress in ALS, particularly driven by mutant superoxide dismutase 1 (SOD1), not only reflects mitochondrial dysfunction but also actively reshapes MAMs [36]. Disruption of MAMs impairs metabolic coupling between the ER and mitochondria, leading to reduced oxidative phosphorylation efficiency and further accumulation of ROS. Importantly, ROS in turn promotes protein aggregation, while aggregates exacerbate oxidative stress through multiple mechanisms, including chaperone exhaustion and interference with MAM integrity [182,184,185]. This means that, in ALS, MAM dysfunction links protein aggregation to oxidative stress and mitochondrial failure, forming a self-amplifying pathogenic loop.

Among the best-characterized contact-site machineries relevant to ALS is the VAPB–PTPIP51 tether, as VAPB itself is an ALS-linked protein and loss of VAPB function has broad consequences for membrane contact site organization and downstream cellular homeostasis [182,186]. Mechanistically, several ALS/FTD-linked pathogenic factors funnel into the disruption of the VAPB–PTPIP51 tether. In Chromosome 9 Open Reading Frame 72 (C9orf72)-associated ALS/FTD, patient-derived neurons and mutant transgenic mice show reduced VAPB–PTPIP51 association together with impaired IP3 receptor–VDAC1 coupling, indicating defective ER-to-mitochondria Ca^2+^ signaling [187]. Importantly, this abnormality is already present before overt disease onset in vivo, supporting its pathogenic relevance at an early stage. Neurotoxic C9orf72-derived dipeptide repeat proteins further impair VAPB–PTPIP51 binding and activate glycogen synthase kinase 3 beta (GSK3β), providing a plausible molecular route by which repeat expansions destabilize ER–mitochondria contacts [187]. Human relevance is strengthened by post-mortem ALS spinal cord analyses showing reduced VAPB levels and decreased VAPB–PTPIP51 proximity in motor neurons, directly demonstrating that ER–mitochondria tether disruption occurs in patient tissue [181]. More broadly, recent ALS-focused reviews synthesize these findings into a model in which dysfunctional mitochondria–ER contacts represent a shared organizing principle across genetically diverse forms of ALS/FTD and highlight the restoration of ER–mitochondria signaling as a potential therapeutic strategy [182,183,188].

### 5.2. Stroke and Ischemia

Ischemic stroke and subsequent reperfusion injury profoundly disrupt inter-organelle communication, positioning membrane contact sites as critical determinants of neuronal and glial fate under acute metabolic stress. Among these, MAMs emerge as a central hub integrating Ca^2+^ signaling, mitochondrial metabolism, autophagy, and cell death pathways during cerebral ischemia/reperfusion injury (CIRI) [25,189].

Acute ischemia induces severe energy depletion, cytosolic Ca^2+^ overload, and oxidative stress, which converge on the endoplasmic reticulum to activate the unfolded protein response (UPR). While transient ER stress may initially promote cellular adaptation, sustained ER stress drives apoptotic signaling cascades that exacerbate ischemic brain injury [190]. Consistent with this, experimental middle cerebral artery occlusion (MCAO) models reveal marked structural alterations in MAMs, including reduced ER-mitochondria contact coverage and increased inter-organelle distance [139]. Such disruption impairs Ca^2+^ homeostasis and mitochondrial function, leading to excessive ROS production and the activation of autophagy-associated cell death pathways [139].

In parallel, ischemic injury can also induce maladaptive reorganization of ER–mitochondria signaling, even in the absence of direct alterations in canonical MAM tethering complexes [191]. The ER-resident protein RTN1-C is markedly upregulated following ischemia/reperfusion and promotes neuronal apoptosis by amplifying ER stress and mitochondria-associated apoptotic pathways. Mechanistically, RTN1-C interacts with the anti-apoptotic protein Bcl-xL, redistributing it to the ER and attenuating its protective function at mitochondria, thereby increasing neuronal vulnerability to ischemic injury [192].

Recent studies further identify specific MAM-associated molecular complexes that actively drive ischemia-induced neuronal death [193]. Pancortin isoforms form complexes with Bcl-xL and WASP family verprolin-homologous protein 1 (WAVE1), localize to MAMs, and potentiate IP_3_ receptor-dependent Ca^2+^ transfer from the ER to the mitochondria. This excessive Ca^2+^ flux leads to mitochondrial Ca^2+^ overload and cell death in models of neonatal and adult ischemic stroke, highlighting MAMs as active execution platforms rather than passive structural features [193].

Importantly, MAM remodeling can be bidirectional and also involves the loss of specific tethering [139]. For example, VAPB and PTPIP51 form a stabilizing tether that preserves ER-mitochondria coupling under physiological conditions. Following ischemic insult, downregulation of the VAPB-PTPIP51 complex compromises MAM integrity, enhances autophagy activation, and worsens neurological outcomes. Conversely, preservation of this tether attenuates infarct volume and neurological deficits, in part through activation of the phosphoinositide 3-kinase (PI3K)/protein kinase B (AKT)/mechanistic target of rapamycin (mTOR) signaling pathway, identifying the VAPB-PTPIP51 axis as an endogenous neuroprotective mechanism [139].

Beyond neurons, glial MAMs play distinct roles in shaping post-ischemic responses. Ischemia-induced remodeling of microglial MAMs promotes maladaptive immunometabolic reprogramming, contributing to sustained neuroinflammation and secondary neuronal injury [23]. In contrast, astrocytes exhibit a reparative mode of MAM plasticity: following ischemic or traumatic injury, ER-mitochondria contacts are enriched in perivascular endfeet, supporting Ca^2+^ buffering, metabolic adaptation, and angiogenic signaling required for vascular remodeling and tissue recovery [59,176].

Together, these findings support a model in which acute brain injury rewires neuron- and glia-specific MCS networks in fundamentally different ways: neuronal MAMs predominantly act as stress-amplifying execution interfaces, whereas glial MAMs participate in inflammatory modulation and tissue repair, ultimately shaping neurodegeneration and functional outcomes after brain injury.

### 5.3. Traumatic Brain Injury

Consistent with ischemic injury, traumatic brain injury (TBI) has been shown to induce changes in ER-mitochondria coupling [194]. In a rodent TBI model, MAM formation was significantly enhanced within the first 24 h post injury, and this enhancement closely correlated with increases in the expression of Ca^2+^ regulatory proteins IP_3_R1, GRP75, and VDAC1, as well as S1R [142]. Production of ROS, levels of ER-stress and UPR response, and release of proinflammatory cytokines were also elevated [142]. Furthermore, attenuation of ER-mitochondrial coupling by silencing PACS2 (phosphofurin acidic cluster sorting protein 2), an ER-mitochondria tethering factor, significantly improved the neurological function of these mice [142]. These findings suggest that dysfunctional MAMs might be primarily involved in neuronal deaths and neurological deficits. However, most evidence comes from rodent and cellular models and direct confirmation in human TBI remains limited.

Interestingly, the MAM responses in TBI seem to have a close relationship with the AD disease process. A study using a moderate cortex impact model found that the MAM-related lipid metabolism is increased in injured cortex, including the C99 accumulation, phosphatidylserine synthesis, and lipid turnover, specifically notable in microglial cells [195]. Relatively limited impairment of mitochondrial respiration was found during the early period examined, but persistent C99 accumulation, ceramide production, and cholesteryl-ester storage created an AD-like lipid environment and contributed to chronic inflammation or neurodegenerative remodeling. Thus, TBI-associated MAM upregulation may initially support membrane repair while becoming maladaptive if sustained [195].

Taken together, these findings suggest that MAM remodeling after TBI is a dynamic process rather than a uniformly detrimental response. The acute enhancement of ER-mitochondria communication may initially support metabolic adaptation and membrane repair, whereas persistent or excessive coupling can disrupt Ca^2+^ and redox homeostasis, activate ER-stress and inflammatory pathways, and promote the other neurological disease, highlighting the importance of distinguishing adaptive from maladaptive inter-organelle communication following injury.

### 5.4. Tumor

In glioblastoma (GBM), mitochondrial dysfunction and cytoskeletal abnormalities are recurrent pathological hallmarks, and emerging evidence indicates that these alterations are functionally interconnected through disease-driven remodeling of ER-mitochondria contact sites [196]. Comparative analyses between glioma stem-like cells (GSCs) and differentiated glioma cells demonstrate marked differences in the abundance of ER-mitochondrial contacts, linking contact-site architecture to tumor cell differentiation state and immune susceptibility. Enforced enhancement of ER-mitochondria contacts reprograms mitochondrial morphology and modifies cell-surface sialylated glycan expression, thereby reducing sensitivity to cytotoxic lymphocyte-mediated killing, highlighting MAMs as regulators of tumor-immune interaction in GBM [197].

Beyond immune modulation, MAM-associated pathways also contribute to the cellular adaption that can support tumor growth and progression [198]. Transcriptomic meta-analyses of glioma specimens further reveal coordinated dysregulation of cytoskeleton-associated and MAM-related genes in high-grade GBM, supporting a functional coupling between organelle tethering and cytoskeletal remodeling during tumor progression [196]. In particular, a meta-analysis found that CLIMP-63 emerged as a multifunctional ER protein linking ER-mitochondrial contact sites, cytoskeletal organization, and oncogenic signaling pathways, with its interaction partner VCP-interacting membrane protein (VIMP) potentially integrating mitochondrial, ER, and cytoskeletal dynamics in glioblastoma progression [196].

It is important to recognize that therapeutic stress further reshapes contact-site networks. In response to temozolomide (TMZ), GBM cells can establish mitochondria-nucleus contact sites in a translocator protein (TSPO)-dependent manner, promoting pro-survival transcriptional programs and contributing to chemotherapy evasion [199]. Importantly, recent mechanistic evidence implicates MAM remodeling in TMZ resistance [200,201,202]. Studies demonstrate that the ER-resident oxidoreductase thioredoxin-related transmembrane protein 1 (TMX1) maintains MAM homeostasis in glioma cells [192]. High TMX1 expression enhances ER-mitochondria communication, supports mitochondrial bioenergetic efficiency, and promotes tumor growth and chemoresistance. Pharmacological targeting of TMX1 using the natural compound Melodinine J disrupts TMX1-dependent MAM integrity, interferes with TMX1-calnexin (CANX) interaction, reduces ER-mitochondria Ca^2+^ flux, impairs oxidative phosphorylation, and induces mitochondrial dysfunction-dependent apoptosis in TMZ-resistant glioma cells [200]. These findings establish TMX1-mediated MAM homeostasis as a structural and metabolic determinant of glioma malignancy.

Taken together, accumulating evidence indicates that disease-driven remodeling of membrane contact sites in GBM integrates cytoskeletal reorganization, metabolic rewiring, immune evasion, and therapy resistance. MAMs therefore represent not merely structural interfaces, but adaptive signaling platforms that coordinate tumor cell survival under oncogenic and chemotherapeutic stress.

## 6. Therapeutic Implications and Challenges

Recent studies highlight membrane contact sites as attractive therapeutic targets in neurological diseases [203,204,205]. However, the pronounced cell-type-, subcellular-, and disease-specific specialization of MCSs poses fundamental challenges for therapeutic intervention. Compared with other classes of MCSs, the molecular composition, regulatory mechanisms, and disease relevance of ER–mitochondria contacts have been investigated in substantially greater detail, resulting in a larger number of experimentally validated therapeutic targets. Accordingly, here we focus on ER-mitochondria contacts a representative and comparatively well-established platform for discussing therapeutic modulation of inter-organelle communication.

### 6.1. Opportunities

Since the complexes anchoring at MCSs are associated with a variety of cellular physiological and pathological phenotypes, they have attracted increasing attention as potential therapeutic targets [204]. The disease mechanisms discussed above collectively indicate that alterations in ER-mitochondria contact sites are not merely secondary consequences of neuronal dysfunction, but actively participate in disease initiation and progression [154]. Across neurodegenerative and ischemic conditions, both excessive tightening and pathological loosening of ER-mitochondria contacts disrupt the finely tuned spatial control of inter-organelle communication, thereby converting a physiological metabolic hub into a driver of cellular vulnerability [139,190,192].

This mechanistic positioning of MAMs as upstream regulators provides a conceptual framework for therapeutic intervention. Rather than targeting isolated downstream effectors—such as Ca^2+^ overload, mitochondrial dysfunction, or protein aggregation—modulation of ER-mitochondria interfaces offers the possibility of restoring multiple dysregulated pathways simultaneously. Importantly, experimental evidence from disease models demonstrates that MAMs’ architecture and function remain plastic and, in several contexts, reversible, supporting the feasibility of pharmacological or genetic interventions aimed at rebalancing ER-mitochondria coupling [105,147].

Moreover, disease-specific mechanisms described in the preceding section directly inform therapeutic directionality. In AD models, in which upregulated MAM activity enhances amyloidogenic APP processing, attenuation of MAM assembly reduces axonal Aβ generation, thereby linking contact site modulation to disease-relevant proteolytic events [147]. Conversely, in ischemic brain injury, pathological reinforcement of MAMs amplifies ER-to-mitochondria Ca^2+^ transfer and neuronal death, and disruption of MAM-associated protein complexes confers neuroprotection [193]. In contrast, reduced ER–mitochondria coupling has been reported in neurodegenerative conditions such as ALS, where pathological TAR DNA-binding protein 43 (TDP-43) disrupts the VAPB–PTPIP51 tethering complex, leading to impaired ER–mitochondria associations and dysregulated Ca^2+^ homeostasis [36,206]. This indicates that loss of contact site integrity can be equally detrimental, highlighting the necessity of restoring, rather than simply inhibiting, inter-organelle coupling in specific disease contexts.

Together, these findings underscore that therapeutic strategies targeting ER-mitochondrial contacts must be context-dependent, aiming not for uniform inhibition or enhancement but for the restoration of physiological inter-organelle communication.

Several studies demonstrate that chemical or genetic modulation of MAMs can directly alter disease-relevant molecular processes. In AD models, downregulation of MAM assembly—either by RNA interference or pharmacological manipulation of MAM-resident proteins such as the S1R—attenuates β-secretase cleavage of palmitoylated APP and reduces amyloid-β generation, particularly within axons, highlighting MAMs as spatially restricted regulators of pathogenic APP processing [147]. These findings support the concept that fine-tuning ER-mitochondria coupling, rather than globally suppressing amyloidogenic enzymes, may offer a more targeted therapeutic avenue with reduced off-target effects.

In addition to MAM-resident proteins directly involved in ER-mitochondria coupling, emerging evidence suggests that pharmacological stabilization of membrane-associated trafficking complexes can also influence neurodegenerative disease progression [207,208]. R55 and R33, two thiopenthene thiourea compounds, have been shown to enhance the stability of the VPS35-VPS29 complex and have the potential to regulate AD progression [209]. Gene network analysis revealed that members of the VPS13-like protein family display differential responses following R55 administration in the mouse brain. Notably, treatment with either R55 or R33 alters the VPS13-like protein family predominantly through functional modulation rather than changes in expression levels, highlighting that small-molecule stabilization of membrane-associated protein complexes can reshape intracellular membrane dynamics and disease-relevant pathways without overt transcriptional reprogramming [209].

In the context of ischemic brain injury, recent work has identified MAMs as critical amplifiers of pathological Ca^2+^ transfer from the ER to mitochondria [193,210]. A2-pancortins promote excessive MAM formation through interactions with Bcl-xL and WAVE1, leading to IP3 receptor-dependent mitochondrial Ca^2+^ overload and neuronal death in neonatal stroke models [193]. Genetic ablation of A2-pancortins or inhibition of ER-to-mitochondria Ca^2+^ flux significantly mitigates ischemic damage, directly implicating MAM-associated protein complexes as actionable targets for neuroprotection [193]. In TBI, the administration of the S1R agonist PRE-084 after disease onset reduced lesion volume, brain edema, microglial activation, and oxidative and nitrosative protein damage while improving neurological recovery [150]. A later study showed that S1R and GRP78 were upregulated after TBI [151]. S1R activation reduced PERK- and IRE1-associated ER stress, mitochondrial ROS, apoptosis, pyroptosis, inflammatory signaling, brain edema, and cerebrovascular dysfunction [151]. These findings give S1R a positive role in stress regulation, but because neither study directly quantified the contact number or contact width after PRE-084 treatment, it remains uncertain whether the protection resulted from structural normalization of MAMs, modulation of MAM-associated signaling, or both.

Beyond disease-specific mechanisms, pharmacological surveys reveal that multiple existing compounds can indirectly modulate MAM structure or function by targeting resident tethering proteins, Ca^2+^ channels, lipid-metabolizing enzymes, or stress-response factors [105]. These studies emphasize that MAMs are highly dynamic and tissue-specific structures, suggesting that therapeutic efficacy will depend on cell type, disease stage, and the precise mode of MAM remodeling [105]. Consistent with this notion, drug screening outcomes have shown that β2-adrenergic receptor signaling promotes ER–mitochondria coupling via defined downstream effectors, revealing G protein-coupled receptor (GPCR) pathways as dynamic regulators of inter-organelle contact formation in response to physiological and stress conditions [205]. Importantly, dysregulated ER-mitochondria contacts have been documented across AD, PD, and ALS/FTD, reinforcing the idea that restoring balanced inter-organelle communication may represent a converging disease-modifying strategy rather than a symptomatic intervention [206].

Finally, emerging evidence linking MAMs to apoptotic signaling and inflammation further broadens their therapeutic relevance [137]. BCL-2 family proteins localized at MAMs not only regulate mitochondrial apoptosis but also influence Ca^2+^ signaling, autophagy, and innate immune activation, positioning ER-mitochondria interfaces as integrative “health meters” of neuronal stress responses [211]. Targeting these nodes may therefore allow coordinated modulation of cell survival and inflammatory pathways in neurodegenerative conditions.

Although our discussion of therapeutic implications primarily focused on MAMs, given their extensive characterization in the context of disease pathogenesis and progression, other contact sites also provide therapeutic insights and opportunities for neurological diseases. For example, regulation of amino acid homeostasis at the mitochondria-lysosome interface has been proposed as a potential targeting axis for PD; the pharmacological regulator 1-phosphatidylinositol 3-phosphate 5-kinase inhibitor can restore Ca^2+^ and lipid metabolism at ER-lysosome interface, which has been tested for ALS treatment [204,212]. Likewise, modulation of SOCE pathways and STIM/ORAI signaling has been proposed as an indirect strategy to influence ER-PM communication, while direct therapeutic targeting of ER–PM contact sites is limited [79].

### 6.2. Risks: Context-Dependent and Cell Type-Specific Effects

Despite the emerging therapeutic potential of targeting ER-mitochondria contact sites, several intrinsic features of these interfaces impose substantial challenges. As discussed in the disease mechanism section, MCSs are highly dynamic, heterogeneous, and strongly dependent on cell type, subcellular compartment, and pathological context. Both excessive tightening and pathological loosening of ER-mitochondrial contacts have been reported in neurological diseases, indicating that MAM dysfunction reflects deviations from physiological homeostasis rather than a unidirectional structural change [206]. Consequently, indiscriminate enhancement or suppression of organelle coupling is unlikely to be therapeutically beneficial and may disrupt essential homeostatic functions.

Moreover, MCSs serve dual roles as adaptive signaling hubs under physiological or transient stress conditions and as maladaptive amplifiers of Ca^2+^ dysregulation, metabolic failure, and cell death under chronic or severe stress [2]. This functional duality suggests that therapeutic efficacy will critically depend on disease stage, timing, and the magnitude of intervention, with a narrow window separating protective modulation from detrimental interference. In addition, pronounced cell-type- and compartment-specific differences—such as those between neurons and glial cells, or between axonal and somatic MAMs—raise concerns about off-target effects and limit the feasibility of broadly acting pharmacological strategies.

Finally, although MCSs integrate multiple pathways implicated in neurodegeneration and ischemic injury, this does not imply that restoring MCS function constitutes a universal therapeutic strategy across neurological diseases. Rather, their role should not be interpreted as a uniform or catch-all mechanism. MCSs provide a spatial framework within which distinct disease-specific molecular programs converge. A major challenge for future therapeutic development is to identify context-selective targets within MCS-associated protein complexes that are preferentially engaged under pathological conditions, while preserving the essential physiological functions of inter-organelle communication.

## 7. Limitation and Open Questions

Despite rapid progress in defining the molecular architecture and functional consequences of MCSs in the brain, several limitations remain to be addressed. First, most of the direct evidence and mechanistic studies in this review come from neurons, whereas evidence in glial cells, especially oligodendrocytes and ependymal cells, remains very limited. Second, the quantitative characterization of MCSs remains insufficient, and comparisons of key physical parameters such as gap distance, contact area, and duration across different cell types and models are limited. Furthermore, the current literature has largely focused on individual MCSs, especially MAMs, and evidence for and mechanisms for the potential relationship and interactions between multiple classes of MCSs remain unclear. Meanwhile, the cell subtype-specific and species-specific differences in molecular organization and regulation require further investigation. Finally, although considerable work has been done to summarize the disease-associated alterations of MCSs, causal relationships between MCSs remodeling and disease initiation and progression require further evidence. Determining whether these changes are the cause or secondary consequences of the disorders will require more investigation. Also, some fundamental questions remain unresolved. Addressing these issues will be essential to move the field from descriptive characterization to the establishment of predictive and therapeutic frameworks.

### 7.1. Expanding the Membrane Contact Site Landscape?

Throughout this review, we have discussed the major well-established MCSs in the brain. However, as the spectrum of MCSs continues to expand, some important questions remain. Future studies should not only identify additional MCSs and their physiological functions, but also uncover the fundamental principles that govern the assembly and regulation of established contact sites.

The context-dependent role of MFN2 exemplifies the latter challenge. As discussed above, the role of MFN2 remains difficult to resolve, as it is reported to either promote or restrict MAMs, likely depending on the cellular context and experimental conditions. Rather than being mutually exclusive, these apparently conflicting findings may reflect a common principle: MFN2 dynamically regulates organelle contacts to meet metabolic demands and cellular responses under pathological conditions. Defining the mechanisms that govern this context dependence will provide a broader framework for understanding how membrane contact sites coordinate inter-organelle communication.

In parallel, the discovery of previously unrecognized MCSs will likely expand the current landscape of organelle communication. One particularly intriguing example is the mitochondria–nucleus contact [213]. These putative contact sites have been proposed to facilitate localized phospholipid metabolism and lipid exchange between the nucleus and mitochondria [214]. They may also play a role in stress adaptation. Neurons rely heavily on oxidative phosphorylation, and therefore probably also rely on the coordination between mitochondria and nucleus to sustain ATP production, redox balance, and mitochondrial integrity. This communication is mediated by mitochondrial metabolites and stress signals, including changes in ATP/AMP and NAD^+^/NADH ratios, reactive oxygen species (ROS), and tricarboxylic acid (TCA) cycle intermediates [213]. Although current evidence for bona fide nuclear–mitochondrial membrane contact sites is largely restricted to non-neuronal systems, recent studies have also identified the mitochondria-nucleus as a central axis of MCSs in AD [215]. Whether this bidirectional communication is spatially organized through specialized MCSs in neurons or glial cells remains unknown and represents an important direction for future investigation. As additional contact sites are identified and their physiological and pathological roles become better understood, future therapeutic development should not remain restricted to the relatively well-characterized classes of MCSs, as these currently under-characterized MCSs may also emerge as promising therapeutic targets.

### 7.2. Distinguishing MCS Remodeling: Adaptive Versus Maladaptive?

One unresolved issue is how to distinguish adaptive from maladaptive remodeling of MCSs. Numerous studies reviewed here demonstrate that both increased and decreased ER-mitochondria coupling can be observed across neurological diseases, including AD, PD, ischemic injury, and glioblastoma [206]. This bidirectionality strongly suggests that the pathological impact of MCS remodeling cannot be inferred solely from changes in contact abundance or ultrastructural proximity.

One key open question is whether there is a physiological ‘operating range’ of MCS coupling that supports metabolic flexibility and stress adaptation, beyond which contact remodeling becomes detrimental. For example, transient MAM tightening may enhance mitochondrial Ca^2+^ uptake and ATP production during short-term stress, whereas chronic or excessive coupling may lead to mitochondrial Ca^2+^ overload, oxidative stress, and apoptosis. Conversely, loosening of contacts may initially protect mitochondria from Ca^2+^ toxicity but ultimately compromise bioenergetic capacity and organelle quality control.

Future studies should move beyond static measurements of contact abundance and instead examine the temporal dynamics, stimulus dependence, and reversibility of MCS remodeling. This will require longitudinal in vivo imaging approaches, genetically encoded reporters of inter-organelle distance and flux, and perturbation strategies that allow graded and reversible modulation of specific contact interfaces rather than the complete ablation of tethering proteins.

### 7.3. Specific Windows for MCS Intervention?

Another major unresolved question concerns the timing of MCS remodeling during disease progression. Increasing evidence suggests that MCS alterations occur early, sometimes preceding overt neurodegeneration, synaptic loss, or inflammatory activation [216]. However, whether these early changes represent compensatory responses or the initial drivers of pathology remains unclear. This raises the possibility that therapeutic windows for targeting MCSs may be highly stage dependent. Interventions that are protective at prodromal or early disease stages may become ineffective or even harmful once downstream degenerative cascades are engaged. Moreover, disease stage may intersect with cell state, particularly in glial cells, where reactive astrocytes and activated microglia exhibit profound metabolic and transcriptional reprogramming that likely reshapes MCS architecture and function.

A related question is whether different neuronal or glial subtype populations display differential sensitivity to MCS perturbation over time. Selective vulnerability in neurodegenerative diseases may therefore not only reflect intrinsic neuronal properties, but also differences in the ability of specific cell types to adaptively remodel MCSs under stress. However, current knowledge of MCS organization and dynamics, especially in glial cells, remains relatively limited, which restricts our ability to determine whether such cell-type-specific differences exist. Resolving this issue will require cell-type- and disease stage-specific in vivo analyses, ideally combined with single-cell or spatial omics approaches linked to ultrastructural data.

### 7.4. Target MCS Functions or Structures?

A critical challenge for the field is whether therapeutic strategies should aim to modulate the physical structure of MCSs or instead target their functional consequences such as Ca^2+^ flux, lipid transfer, redox signaling, or organelle quality control. Given the pleiotropic roles of MCSs and their strong context dependence, direct manipulation of tethering complexes may carry substantial risk of off-target effects. In contrast, selectively tuning downstream signaling nodes—such as IP_3_R-mediated Ca^2+^ release, mitochondrial Ca^2+^ uptake thresholds, lipid transfer capacity, or stress-responsive chaperone activity—may offer greater specificity while preserving essential basal MCS functions.

An emerging point is that MCSs act more as dynamic signaling platforms whose functional output depends on local protein composition, lipid environment, and biophysical properties such as membrane curvature and phase separation. Understanding how these properties are regulated, and how they differ between neurons and glial cells, will be essential for identifying intervention points that decouple pathological signaling from physiological inter-organelle communication. Furthermore, successful translation of MCS-targeted drugs will likely require not only precise identification of signaling nodes, but also effective delivery strategies. Although beyond the primary scope of this review, emerging delivery platforms such as extracellular vesicles may facilitate future translation of MCS-targeted therapeutics by enabling more selective delivery of molecular interventions to the nervous system.

### 7.5. Toward Integrative and Predictive Models of Brain MCSs

Finally, a major future direction is to integrate structural, molecular, and functional data into predictive models of MCS behavior in the brain. At present, most studies examine individual contact sites, tethering proteins, or signaling pathways in isolation. However, neurons and glial cells harbor complex, interconnected networks of MCSs that likely compete for shared resources and coordinate organelle behavior at the systems level. Developing such integrative models will require combining high-resolution imaging, quantitative proteomics, biophysical modeling, and functional readouts across multiple organelles and cell types. Ultimately, a systems-level understanding of MCS networks may reveal emergent properties, such as buffering capacity, signaling redundancy, or failure modes, that cannot be inferred from individual contact sites alone.

## 8. Conclusions

Throughout this review, we summarized the current understanding of MCSs in the central nervous system, with particular emphasis on their cell type-specific organization, subcellular specialization, pathological remodeling, and therapeutic potential. We first highlighted the distinct organization of MCSs across different neural cell types. Although neurons and glial cells—particularly astrocytes and microglia—share many tethering complexes and functional proteins, they exhibit distinct organizational principles and functional preferences. Our analysis suggests that neuronal MCSs are primarily adapted to meet the exceptionally high demands for rapid Ca^2+^ signaling, energy production, and metabolic flexibility, whereas glial MCSs preferentially coordinate lipid metabolism, immune signaling, metabolic support, and tissue homeostasis.

We further examined the spatial specialization of MCSs within neurons, demonstrating that their organization and function are also highly compartment dependent. Somatic MCSs are enriched around large, sheet-like ER cisternae and extensive MAMs, enabling global metabolic regulation and the maintenance of cellular homeostasis. In contrast, dendritic MCSs, particularly those within dendritic spines, are more spatially restricted and specialized for local Ca^2+^ signaling and synaptic plasticity. Presynaptic MCSs are optimized to support activity-dependent neurotransmission and vesicular trafficking, whereas axonal MCSs contribute to mitochondrial transport, organelle quality control, and axonal resilience. These observations indicate that both cell identity and subcellular localization fundamentally shape the architecture and functional output of MCSs.

We also summarized the current evidence demonstrating that MCSs undergo extensive remodeling across a broad spectrum of neurological disorders. Importantly, disease-associated MCS remodeling is not merely characterized by increased or decreased contact abundance, but also involves profound alterations in contact-site composition, molecular organization, functional output, and dynamic behavior. These observations underscore that MCS dysfunction cannot be adequately interpreted by ultrastructural changes alone but instead reflects complex reprogramming of inter-organelle communication.

Finally, we reviewed emerging therapeutic strategies targeting MCSs and found that an increasing number of pharmacological approaches are beginning to directly modulate MCS-associated pathways. Rather than viewing MCSs simply as structural bridges between organelles, current evidence supports the concept that they function as dynamic signaling platforms. Accordingly, future therapeutic strategies may benefit more from selectively modulating MCS-mediated signaling and organelle communication than from indiscriminately increasing or decreasing organelle contacts.

Together, these findings establish MCSs as fundamental organizers of inter-organelle communication whose spatial and molecular specialization underlies both neural homeostasis and disease.

## Figures and Tables

**Table 1:** Major membrane contact site proteins in the nervous system.

Major MCS Pair	Protein/Complex	Principal Physiological Function	Representative Disease Association(s)	Experimental Systems Used	References
ER–mito	IP_3_R–GRP75–VDAC1	Mediates ER-to-mitochondria Ca^2+^ transfer and regulates mitochondrial bioenergetics and Ca^2+^ homeostasis	AD, PD, ALS, ischemic injury, TBI	Mammalian cell lines; primary neurons and glial cells; rodent brain and neurological disease models	[2,3,31,32,33,72,73,94,107,142,143,144,145]
	VAPB–PTPIP51	ER–mitochondria tethering; Ca^2+^ transfer; mitochondrial bioenergetics	AD, PD, ALS/FTD	Mammalian cell lines; primary neurons; glial cells; neuronal disease models, particularly ALS/FTD; mouse tissue, etc.	[16,27,34,35,36,37,38,39,40,139,144,145]
	MFN2	Regulation of ER–mitochondria contacts; mitochondrial fusion; neurite development	AD, PD	Mammalian cell lines; primary neurons; astrocytes, mouse models, etc.	[20,27,44,45,46,47,48,49,59]
	PDZD8	Local ER-to-mitochondria Ca^2+^ transfer; lipid transport	Synaptic dysfunction	HeLa, HEK293T, Neuro2a and other mammalian cells; mouse primary cortical neurons; cortical/hippocampal neurons in vivo; mouse brain; Drosophila neurons, etc.	[27,41,42,43,72,96,97,98]
	GDAP1	Coupling Ca^2+^ transfer to ATP production	Charcot–Marie–Tooth disease	Human SH-SY5Y neuroblastoma cells; primary embryonic motor neurons from WT and Gdap1−/− mice; CMT disease models, etc.	[27,72,146]
	S1R	Regulation of IP_3_R stability; ER stress adaptation; Ca^2+^ signaling	AD, ALS, HD, TBI	Mammalian cell lines; primary neurons; glial cells; rodent brain and neuronal disease models, etc.	[99,101,142,147,148,149,150,151]
	RTN1A	Promotion of ER–mitochondria contacts	Unknown	Neuronal cell lines; primary neurons; rodent nervous tissue, etc.	[104]
	GET4	Regulation of ER–mitochondria contacts and Ca^2+^ dynamics	AD model	Mammalian cell lines; neuronal models are comparatively limited.	[106]
	BAG6	ER membrane quality control; modulation of Ca^2+^ signaling	Unknown	Mammalian cell lines; neuronal models are comparatively limited.	[106]
	REEP1	ER shaping; regulation of ER–mitochondria contacts	HSP	Mammalian cell models; mouse embryonic brain; neuronal/HSP models, etc.	[104,152,153,154]
	Atlastin	organizing mitochondrial distribution and transport machinery, injury repairing	HSP	Mammalian cell lines, etc.	[104,153]
ER-mito/ ER– endosome	Spastin	regulating mitochondrial morphology, calcium homeostasis, and oxidative metabolism; promoting the fission of endosomal tubules, injury repairing	HSP	Mammalian cell lines; human SH-SY5Y neuroblastoma cells; mouse MEF.	[104,153]
	Protrudin	Endosome transport; neurite extension	HSP	Mammalian cell lines; mouse primary cortical neurons; mouse brain.	[87,97,140,141]
ER–PM	ESYT1	ER–PM tethering; synaptic plasticity	AD	Mammalian cell lines; neuronal models are comparatively limited.	[81]
	STIM2–ORAI1	Store-operated Ca^2+^ entry (SOCE)	AD	Hippocampal/cortical neuronal systems; rodent brain models	[27,80,155]
	TMEM24	Lipid transfer	Unknown	Mammalian cell lines; mouse hippocampal neuron.	[114,115]
Mito– lysosome	VAMP7/8	Regulation of mitochondria–lysosome contact dynamics and mitochondrial homeostasis, independently of autophagic fusion	Unknown	Drosophila.	[64]
	Rab7–FIS1–TBC1D15	Contact untethering; mitochondrial dynamics	PD	HeLa and other mammalian cell lines; human iPSC-derived neurons; dopaminergic neuronal models.	[62,63,156,157]
ER–lysosome/ER– late endosome	VPS13C	Lipid transport	PD	Mammalian cell lines; human iPSC-derived neurons; dopaminergic neuronal/PD models.	[116]
ER–lysosome	VAPA/B–OSBP	Cholesterol trafficking; mTORC1 activation	AD	Mammalian cell lines; primary neurons, etc.	[55]

## Data Availability

No new data were created or analyzed in this study. Data sharing is not applicable to this article.

## Abbreviations

The following abbreviations are used in this manuscript:

Aβ	Amyloid-β
Aβo	Amyloid-β oligomers
AD	Alzheimer’s disease
AICD	Amyloid precursor protein intracellular domain
AKAP1	A-kinase anchoring protein 1
ALS	Amyotrophic lateral sclerosis
ALS/FTD	Amyotrophic lateral sclerosis/frontotemporal dementia
AMPA	α-Amino-3-hydroxy-5-methyl-4-isoxazolepropionic acid
APAF-1	Apoptotic protease activating factor 1
APP	Amyloid precursor protein
ARL6IP1	ADP-ribosylation factor-like 6 interacting protein 1
ASC	Apoptosis-associated speck-like protein containing a CARD
ATF4	Activating transcription factor 4
ATL	Atlastin
BACE1	β-Site APP-cleaving enzyme 1
BAG6	BCL2-associated athanogene 6
BCL2L13	BCL2-like 13
BDH2	3-Hydroxybutyrate dehydrogenase 2
BiP	Binding immunoglobulin protein
CaV1	Voltage-gated L-type calcium channel 1
CARD	Caspase recruitment domain
Cav1–RyR2–KCa3.1	Voltage-gated L-type calcium channel 1–Ryanodine receptor 2–Calcium-activated potassium channel 3.1 complex
CHOP	C/EBP homologous protein
CIRI	Cerebral ischemia–reperfusion injury
CLIMP63	Cytoskeleton-linking membrane protein 63
DRP1	Dynamin-related protein 1
ER	Endoplasmic reticulum
ESCRT	Endosomal sorting complexes required for transport
ESYT1	Extended synaptotagmin 1
FAM134B	Family with sequence similarity 134 member B
FICD	Filamentation induced by cAMP domain-containing protein
FIS1	Mitochondrial fission 1 protein
GalCer	Galactosylceramide
GBA1	Glucosylceramidase beta 1
GCase	β-Glucocerebrosidase
GDAP1	Ganglioside-induced differentiation-associated protein 1
GET4	Guided entry of tail-anchored proteins factor 4
GBM	Glioblastoma
GLTP	Glycolipid transfer protein
GPCR	G protein-coupled receptor
GPX4	Glutathione peroxidase 4
GPR158	G protein-coupled receptor 158
GRP75	Glucose-regulated protein 75
GRP78	78 kDa glucose-regulated protein
GSCs	Glioblastoma stem cells
GSK3β	Glycogen synthase kinase 3 beta
HDAC2/3	Histone deacetylase 2/3
hiPSCs	Human induced pluripotent stem cells
HSP	Hereditary spastic paraplegia
HSP70	Heat shock protein 70
IDR	Intrinsically disordered region
IP3R	Inositol 1,4,5-trisphosphate receptor
IP3R–GRP75–VDAC	Inositol 1,4,5-trisphosphate receptor–Glucose-regulated protein 75–Voltage-dependent anion channel complex
iPLA2-VIA	Calcium-independent phospholipase A2 group VIA
IRE1	Inositol-requiring enzyme 1
ISC	Iron–sulfur cluster
KCa3.1	Intermediate-conductance calcium-activated potassium channel 3.1
Kv2.1	Voltage-gated potassium channel Kv2.1
LC3B	Microtubule-associated protein 1 light chain 3 beta
LD	Lipid droplet
LTP	Long-term potentiation
LXRα	Liver X receptor alpha
MAM	Mitochondria-associated membrane
MAVS	Mitochondrial antiviral-signaling protein
MCAO	Middle cerebral artery occlusion
MCS	Membrane contact site
MCU	Mitochondrial calcium uniporter
Mfn2	Mitofusin 2
Miro1	Mitochondrial Rho GTPase 1
mTORC1	Mechanistic target of rapamycin complex 1
NMDA	N-Methyl-D-aspartate
NLRP3	NOD-, LRR-, and pyrin domain-containing protein 3
Nrf2	Nuclear factor erythroid 2-related factor 2
OSBP	Oxysterol-binding protein
PACS2	Phosphofurin acidic cluster sorting protein 2
PD	Parkinson’s disease
PDZD8	PDZ domain-containing protein 8
PERK	Protein kinase R-like endoplasmic reticulum kinase
PI4P	Phosphatidylinositol 4-phosphate
PINK1	PTEN-induced kinase 1
PIP_2_	Phosphatidylinositol 4,5-bisphosphate
PKA	Protein kinase A
PLA2G6	Phospholipase A2 group VI
PLCXD2	Phosphatidylinositol-specific phospholipase C X domain-containing protein 2
PM	Plasma membrane
PRRs	Pattern recognition receptors
PS	Phosphatidylserine
PTPIP51	Protein tyrosine phosphatase-interacting protein 51
PUFA	Polyunsaturated fatty acid
RAB7	Ras-related protein Rab-7
REEP1	Receptor expression-enhancing protein 1
RHOT1	Ras homolog family member T1
ROS	Reactive oxygen species
RRBP1	Ribosome-binding protein 1
RTN1A	Reticulon 1A
RyR	Ryanodine receptor
RyR2	Ryanodine receptor 2
S1R	Sigma-1 receptor
SCD1	Stearoyl-CoA desaturase 1
Sec22b	SEC22 homolog B
SNARE	Soluble N-ethylmaleimide-sensitive factor attachment protein receptor
SOCE	Store-operated calcium entry
SOD1	Superoxide dismutase 1
SPAST	Spastin
SPG3A	Spastic paraplegia type 3A
SPG4	Spastic paraplegia type 4
SPG31	Spastic paraplegia type 31
SPG33	Spastic paraplegia type 33
STIM	Stromal interaction molecule
STX1	Syntaxin 1
TBI	Traumatic brain injury
TBC1D15	TBC1 domain family member 15
TCA	Tricarboxylic acid
TDP-43	TAR DNA-binding protein 43
TMZ	Temozolomide
TMX1	Thioredoxin-related transmembrane protein 1
TRP	Transient receptor potential
TSPO	Translocator protein
UPR	Unfolded protein response
VAMP	Vesicle-associated membrane protein
VAPA	Vesicle-associated membrane protein-associated protein A
VAPB	Vesicle-associated membrane protein-associated protein B
VDAC	Voltage-dependent anion channel
VIMP	VCP-interacting membrane protein
VPS13	Vacuolar protein sorting-associated protein 13
WAVE1	WASP family verprolin-homologous protein 1
XBP1	X-box-binding protein 1
